# LncRNA TUG1 mediates microglial inflammatory activation by regulating glucose metabolic reprogramming

**DOI:** 10.1038/s41598-024-62966-4

**Published:** 2024-05-27

**Authors:** Chunxiang He, Ze Li, Wenjing Yu, Rongsiqing Luo, Jinyong Zhou, Jiawei He, Qi Chen, Zhenyan Song, Shaowu Cheng

**Affiliations:** 1 School of Integrated Chinese and Western Medicine, Hunan University of Chinese Medicine, Changsha, 410208 Hunan China; 2 Key Laboratory of Hunan Province for Integrated Traditional Chinese and Western Medicine On Prevention and Treatment of Cardio-Cerebral Diseases, School of Integrated Chinese and Western Medicine, Hunan University of Chinese Medicine, Changsha, 410208 Hunan China; 3 Office of Science & Technology, Hunan University of Chinese Medicine, Changsha, 410208 Hunan China

**Keywords:** LncRNA TUG1, Microglia, Glucose metabolic reprogramming, Glycolysis, Inflammation, Cell biology, Cytokines, Inflammation, Microglia

## Abstract

Microglia are natural immune cells in the central nervous system, and the activation of microglia is accompanied by a reprogramming of glucose metabolism. In our study, we investigated the role of long non-coding RNA taurine-upregulated gene 1 (TUG1) in regulating microglial glucose metabolism reprogramming and activation. BV2 cells were treated with Lipopolysaccharides (LPS)/Interferon-γ (IFN-γ) to establish a microglial activation model. The glycolysis inhibitor 2-Deoxy-D-glucose (2-DG) was used as a control. The expression levels of TUG1 mRNA and proinflammatory cytokines such as Interleukin-1β (IL-1β), Interleukin -6, and Tumor Necrosis Factor-α mRNA and anti-inflammatory cytokines such as IL-4, Arginase 1(Arg1), CD206, and Ym1 were detected by RT-qPCR. TUG1 was silenced using TUG1 siRNA and knocked out using CRISPR/Cas9. The mRNA and protein expression levels of key enzymes involved in glucose metabolism, such as Hexokinase2, Glyceraldehyde-3-phosphate dehydrogenase (GAPDH), Lactate dehydrogenase, Glucose 6 phosphate dehydrogenase, and Pyruvate dehydrogenase (PDH), were determined by RT-qPCR and Western blotting. The glycolytic rate of microglial cells was measured using Seahorse. Differential metabolites were determined by metabolomics, and pathway enrichment was performed using these differential metabolites. Our findings revealed that the expression of TUG1 was elevated in proinflammatory-activated microglia and positively correlated with the levels of inflammatory factors. The expression of anti-inflammatory cytokines such as IL-4, Arg1, CD206, and Ym1 were decreased when induced with LPS/IFN-γ. However, this decrease was reversed by the treatment with 2-DG. Silencing of GAPDH led to an increase in the expression of TUG1 and inflammatory factors. TUG1 knockout (TUG1KO) inhibited the expression of glycolytic key enzymes and promoted the expression of oxidative phosphorylation key enzymes, shifting the metabolic profile of activated microglia from glycolysis to oxidative phosphorylation. Additionally, TUG1KO reduced the accumulation of metabolites, facilitating the restoration of the tricarboxylic acid cycle and enhancing oxidative phosphorylation in microglia. Furthermore, the downregulation of TUG1 was found to reduce the expression of both proinflammatory and anti-inflammatory cytokines under normal conditions. Interestingly, when induced with LPS/IFN-γ, TUG1 downregulation showed a potentially beneficial effect on microglia in terms of inflammation. Downregulation of TUG1 expression inhibits glycolysis and facilitates the shift of microglial glucose metabolism from glycolysis to oxidative phosphorylation, promoting their transformation towards an anti-inflammatory phenotype and exerting anti-inflammatory effects in BV2.

## Introduction

Microglia, the natural immune cells in the central nervous system (CNS), play a crucial role in nerve development by phagocytosing and clearing damaged neurons and synapses^1^. They are renowned for their protective effects in maintaining CNS homeostasis, acting as the frontline defense of the innate immune system and regulating neuronal populations. However, it is important to highlight that persistent microglial activation has been implicated in the pathogenesis of neurodegenerative diseases such as Alzheimer's disease (AD)^2^. Additionally, microglia are major contributors to neuroinflammation, further emphasizing their role in these processes^3^.

Glycolysis plays a pivotal role in the inflammatory response, and immunometabolism emerges as a central regulator of inflammation, providing new avenues for treating inflammation-related pathologies^4^. Upon activation, immune cells reprogram their metabolic pathways to meet the energy and biosynthesis demands. In the case of lymphocytes, including inflammatory M1 macrophages, there is a shift from oxidative phosphorylation (OXPHOS) to glycolysis, whereas regulatory T cells and M2 macrophages predominantly utilize the tricarboxylic acid (TCA) cycle with reduced reliance on glycolysis^5^. Microglia, similar to peripheral macrophages, exhibit comparable functional and phenotypic changes in response to activation. These changes are accompanied by significant alterations in cell metabolism. Notably, proinflammatory M1 macrophages primarily rely on glycolysis, which enables the production of antimicrobial metabolites like itaconate and succinate, achieved by interrupting the TCA cycle twice. Accumulation of excess succinate promotes the stabilization of HIF1α, which subsequently activates the transcription of genes involved in glycolysis, thereby sustaining M1 metabolic phenotype. This metabolic shift, famously known as the Warburg effect, is characterized by increased glucose uptake, elevated glycolytic flux, pyruvate conversion to lactate, and reduced oxygen consumption even in oxygen-rich environments^6^. Such metabolic adaptations have been observed in microglia as well, highlighting their metabolic plasticity in response to inflammatory cues.

The long non-coding RNA (lncRNA) taurine upregulated gene 1 (TUG1) was initially discovered in the murine retina and has been found to be essential for retina development^7^. Recent studies have implicated TUG1 in various inflammatory responses, such as cardiomyocyte ischemia–reperfusion injury and acute lung injury^8,9^. Additionally, TUG1 has been shown to be upregulated in hepatocyte inflammation induced by lipopolysaccharide (LPS). Silencing TUG1 has been found to alleviate LPS-induced hepatocyte inflammation by targeting the miR-140/TNF pathway^10^. Furthermore, TUG1 has been identified as a potential biomarker for Parkinson's disease, and its downregulation may inhibit the inflammatory response in the progression of the disease^11^. In the context of cancer, TUG1 has been revealed as a crucial component of cancer-associated fibroblast (CAF)-secreted exosomes that promote migration, invasion, and glycolysis in hepatocellular carcinoma (HCC) cells through the miR-524-5p/SIX1 axis^12^. However, the role of TUG1 in the inflammatory response and glucose metabolism of microglia remains poorly understood. In this study, we aimed to investigate the impact of TUG1 on microglial glucose metabolism and activation, shedding light on the underlying mechanisms involved.

## Materials and methods

### Cell culture and treatment

Mouse microglial cells (BV2 Cells) were purchased from Procell Life Science & Technology Co., Ltd. and were maintained in Roswell Park Memorial Institute (RPMI) 1640 supplemented with 10% fetal bovine serum, and 1% penicillin–streptomycin solution in a humidified incubator with 5% CO_2_ at 37 °C. BV2 was treated with LPS (sigma, L2880, 100 ng/ml)/IFN-γ (Pepro Tech, 315–05-100, 10 ng/ml) for 6, 12, and 24 h.

### Cell transfection and CRISPR/Cas9 genetic editing

The siRNA of TUG1 (sense: 5′-CAUCCAAAGUGAACUACGUTT-3′, antisense: 5′-ACGUAGUUCACUUUGGAUGTT-3′) was transfected into cells using Lipofectamine 2000. To knock out TUG1 in BV2 cells, seven sgRNAs were designed, and the sgRNA sequences are listed in Table S1. After testing and screening, the NO.5 TUG1KO BV2 cell line was used for further experiments (Figure S1).

### RT-qPCR

The total RNA was extracted using Trizol reagent and reverse-transcribed into cDNA using the NovoScript 1st Strand cDNA Synthesis SuperMix (gDNA Purge) (novoprotein, E047-01B). cDNA amplification was performed using the MonAmp™ SYBR® Green qPCR Mix (Monad, MQ00401) according to the manufacturer’s instructions on a Bio-Rad CFX96 Real-time PCR system. The primers used for the RT-qPCR are listed in Table 1. The comparative Ct method (2^-ΔΔCt^) was used to analyze the data.

**Table 1:** Primer sequences.

Gene	Forward	Reverse
Arg1	5′-GCAACCTGTGTCCTTTCTCCT-3′	5′-GTCTCTTCCATCACCTTGCCA-3′
CD206	5′-CAAGGAAGGTTGGCATTTGT-3′	5′-CCTTTCAGTCCTTTGCAAGC-3′
GAPDH	5′-AGTCCACCTGGCGTCTTCAC-3′	5′-GAGGCATTGCTGATGATCTTGA-3′
HK2	5′-GCGTGGATGGCTCTGTCTACAAG-3′	5′-GGAGGAAGCGGACATCACAATCG-3′
IL-1β	5′-TCGCAGCAGCACATCAACAAGAG-3′	5′-TGCTCATGTCCTCATCCTGGAAGG-3′
IL-4	5′-AAACGTCCTCACAGCAACGA-3′	5′-CAGCTTATCGATGAATCCAGGCA-3′
IL-6	5′-CTCCCAACAGACCTGTCTATAC-3′	5′-CCATTGCACAACTCTTTTCTCA-3′
LDHA	5′-CAAAGACTACTGTGTAACTGCGA-3′	5′-TGGACTGTACTTGACAATGTTGG-3′
PFKFB3	5′-TTGTCCAGCAGAGGCAAGAAGTTC-3′	5′-CACACACGGAGGTCCTTCAGATTC-3′
PKM2	5′-GTGCCGCCTGGCGAGGGGACTC-3′	5′-TTCAGCCGAGCCACATTCATTCC-3′
TNF-α	5′-GTCTCAGCCTCTTCTCATTCC-3′	5′-CTACAGGCTTGTCACTCGAA-3′
Tug1	5′-CATCTCACAAGGCTTCAACCA-3′	5′-AAGGACTCACTTCTACCACTGT-3′
Ym1	5′-CAGTGTTCTGGTGAAGGAAATG-3′	5′-ACCCAGACTTGATTACGTCAAT-3′
β-actin	5′-AAGTGTGACGTTGACATCCG-3′	5′-TCTGCATCCTGTCAGCAATG-3′

### Western blotting

The total protein was extracted using a total protein extraction buffer premixed with protease and phosphatase inhibitors and quantified using a BCA protein assay kit. Equal amounts of protein (20 μg) were separated by 10% sodium dodecyl sulfate–polyacrylamide gel electrophoresis and transferred onto a polyvinylidene fluoride (PVDF) membrane. The membrane was blocked with 5% fresh nonfat milk in tris buffered saline (containing 0.5‰ tween-20, TBST) at room temperature for 1 h and then incubated with antibodies against HK2 (CST, 28,678, 1:2000), PDH (Abcam, ab110330, 1:2000), G6PD (Abcam, ab993, 1:2000), tubulin (sigma, T5168, 1:8000), β-actin (Affinity, AF7018, 1:8000) overnight at 4 °C. After three washes with TBST, the membranes were incubated with secondary antibody (Elabscience, E-AB-1001/1003, 1:8000) for 1 h at room temperature. The membranes were then visualized using the ChemiDoc XRS + Gel Imaging System (BIO-RAD).

### Glycolytic rate assay

A Glycolytic Rate Assay was performed to assess cellular energy metabolism. Fluorescence-free plates were first coated with 0.1% gelatin and seeded with 10,000 cells per well. The cells were allowed to adhere overnight and induced for 24 h with or without LPS/IFN-γ before being cultured in phenol red-free DMEM containing 10 mM glucose, 1 mM pyruvate, and 2 mM L-glutamine in a non-CO_2_ incubator at 37 °C for 1 h. Plates were then loaded into a Seahorse XFe96 analyzer and subjected to sequential injections of a mixture of rotenone and antimycin A (final concentrations of 0.5 μM) and 2-DG (final concentrations of 50 μM). Real-time measurements were taken to quantify cellular oxygen consumption rates (OCR) and extracellular acidification rates (ECAR). The final OCR and ECAR readings were normalized to the number of cells in each well, as determined by crystal violet staining. All Seahorse experiments included appropriate positive and negative controls and were repeated a minimum of three times for statistical analysis.

### Metabolomics

After the cells were collected, the samples were weighed and transferred into 2 mL centrifuge tubes. Next, 600 μL of 80% acetonitrile–water was added, and the mixture was subjected to grinding for 5 min, vortex mixing for 1 min, ultrasonic oscillation for 30 min, and centrifugation at 4000 rpm for 10 min. The supernatant was collected and stored for further use. Subsequently, 200 μL of the supernatant was transferred into a 1.5 mL centrifuge tube, followed by the addition of 100 μL of 3-NPH (200 mM) and 100 μL of EDC (120 mM; containing 6% pyridine). The solution was vortexed for 1 min and placed in a 40 ℃ water bath for 1 h with occasional shaking. After the reaction was completed, the solution was centrifuged at 12,000 rpm and 4 ℃ for 15 min, and the supernatant was collected and filtered through a 0.22 μm filter before undergoing LC–MS/MS analysis. The sample was diluted 100 times with 80% acetonitrile–water before analysis. Finally, MultiQuant software was used to integrate and calculate the content of the samples using the standard curve^13^.

### Statistical analysis

The statistical analyses were conducted using Graph Pad Prism 8 software. The data are presented as the mean ± SEM. A two-tailed Student’s t-test was used to assess the differences between the two groups, and one-way ANOVA coupled with Sidak’s post-hoc test was used to assess differences among the three groups. *P* < 0.05 was statistically significant.

## Results

### TUG1 was upregulated in microglia induced with LPS / IFN-γ

**Figure 1:**
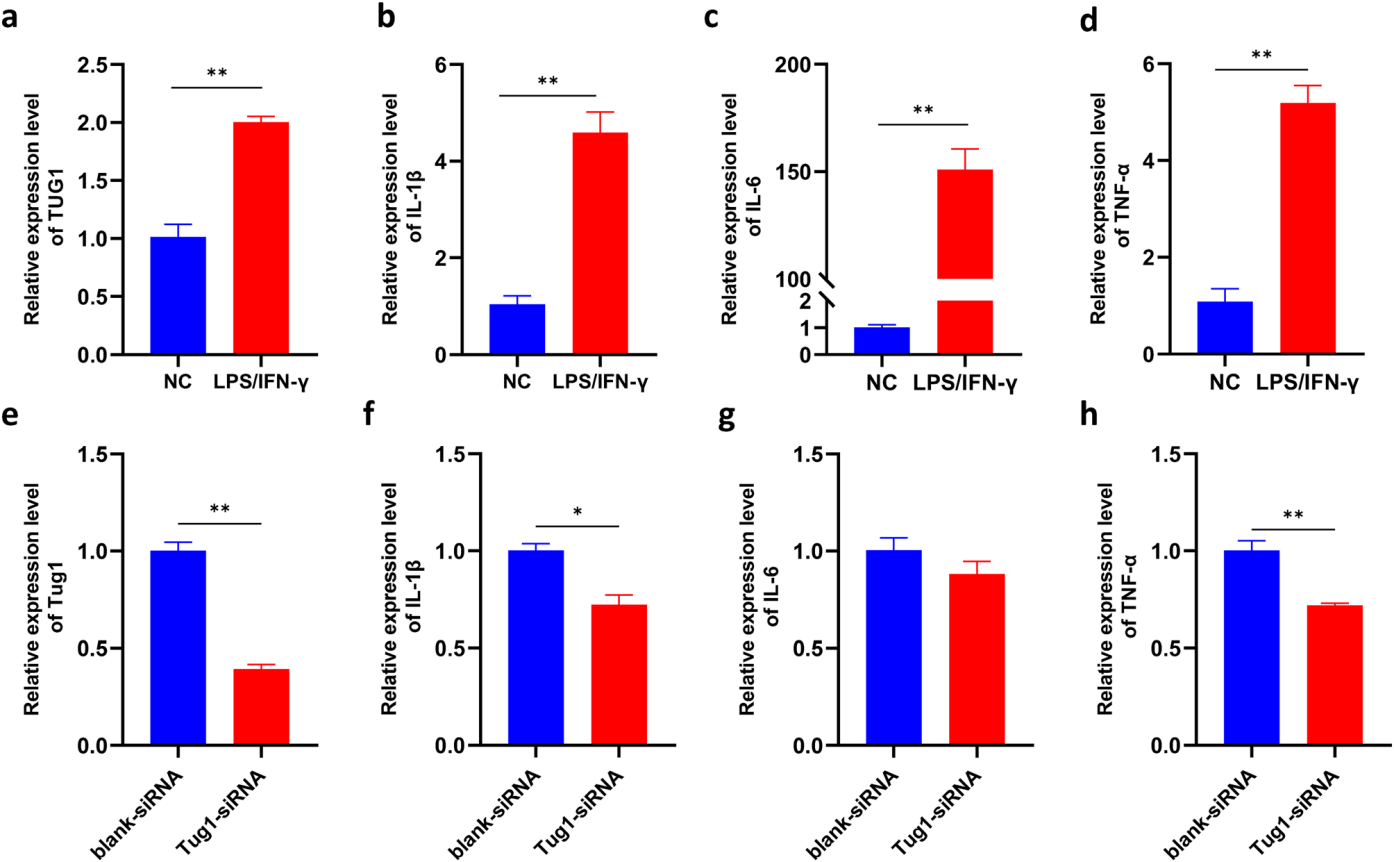
TUG1 is involved in the regulation of glucose metabolism in microglial activation. (**a**) The expression of TUG1 mRNA after induced with LPS (100 ng/ml)/IFN-γ(10 ng/ml) for 24 h (n = 3). (**b**–**d**) The expression of IL-1β,IL-6, and TNF-α mRNA after induced with LPS (100 ng/ml)/IFN-γ(10 ng/ml) for 24 h (n = 3). (**e**) The expression of TUG1 mRNA after transfected with TUG1siRNA (n = 3). (**f**–**h**) The expression of IL-1β,IL-6, and TNF-α mRNA after transfected with TUG1siRNA (n = 3). Error bars indicate SEM. *P < 0.05, **P < 0.01.

Microglia play an important role in inflammation^14^. We wanted to know whether the microglia-mediated inflammation was regulated by TUG1. In vitro, the expression of inflammatory factors IL-1β, IL-6, and TNF-α were also significantly up-regulated in BV2 induced with LPS/IFN-γ when compared to the normal control group (Fig. 1b-d). The expression of TUG1 was consistent with the rising inflammatory factors (Fig. 1a). The results suggest TUG1 may participate the microglia-mediated inflammation.

To further explore the role of TUG1 in microglial activation, we transfected TUG1siRNA to BV2 with Lipofectamine (TM)2000 (Lip2000). The expression of TUG1 was significantly lower than the blank-siRNA group (Fig. 1e). Similarly, the levels of inflammatory factors such as IL-1β, IL-6, and TNF-α were down-regulated in the BV2 after transfected with TUG1-siRNA (Fig. 1f-h). These findings demonstrate a positive correlation between TUG1 expression and the expression of inflammatory factors, suggesting a potential regulatory role of TUG1 in the modulation of inflammation.

### Silencing of GAPDH leads to an upregulation of TUG1 and inflammatory factors expression in microglia

**Figure 2:**
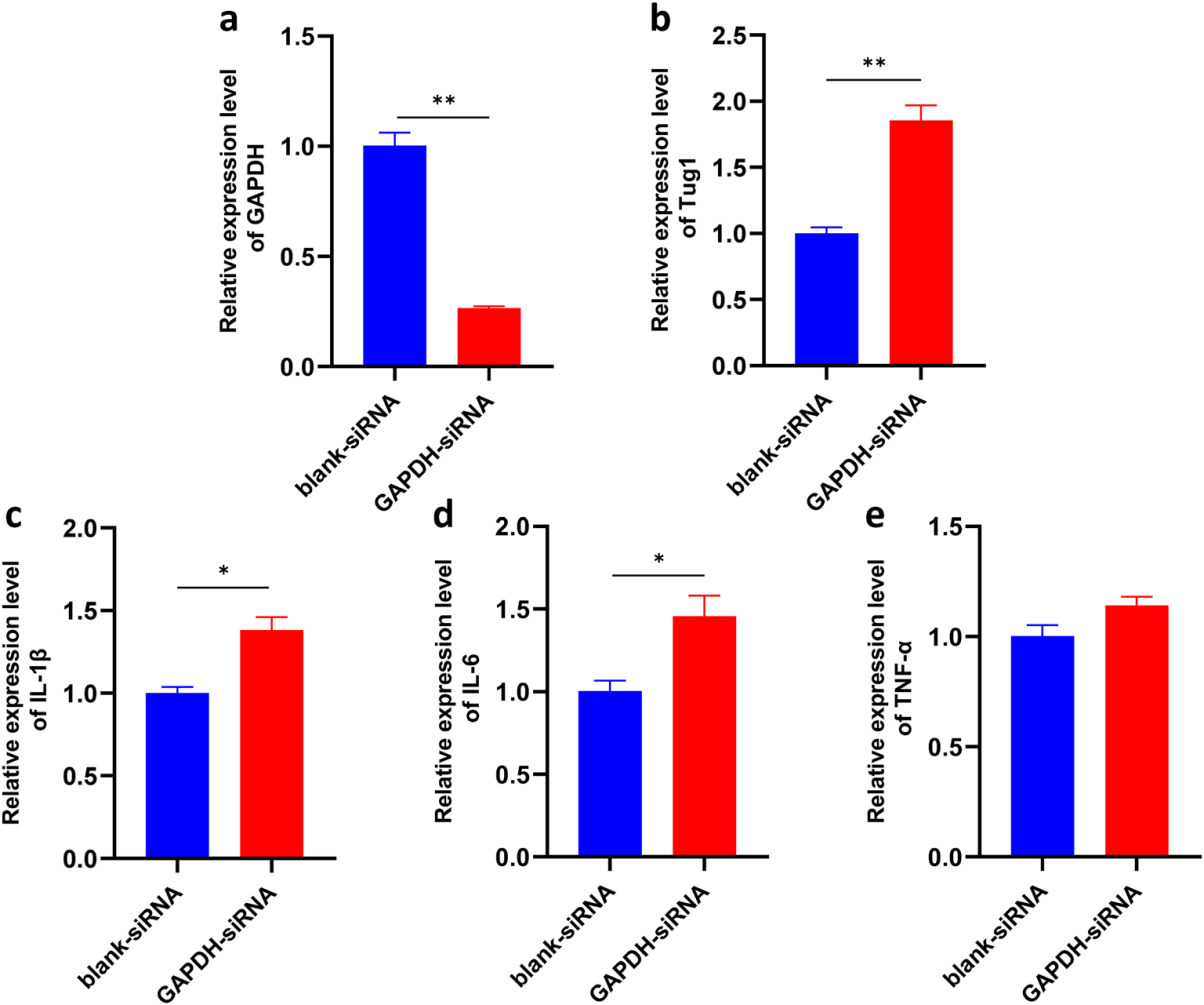
TUG1 is involved in the regulation of glucose metabolism in microglial activation. (**a**–**b**). The expression of GAPDH and TUG1 mRNA after silencing with GAPDH siRNA, respectively (n = 3). (**c**–**e**). The expression of IL-1β, IL-6, and TNF-α mRNA after silencing with GAPDH siRNA, respectively (n = 3). Error bars indicate SEM. *P < 0.05, **P < 0.01.

Further, the silencing of GAPDH is associated with an upregulation of TUG1 expression and an increase in the expression of inflammatory factors IL-1β, IL-6, and TNF-α (Fig. 2a-e). Given the pivotal role of GAPDH, a key enzyme involved in glycolysis^15^, we hypothesize that TUG1 may be involved in the proinflammatory activation of microglial cells by modulating the glucose metabolism pathway.

### TUG1 is involved in the regulation of glucose metabolism in microglial activation

**Figure 3:**
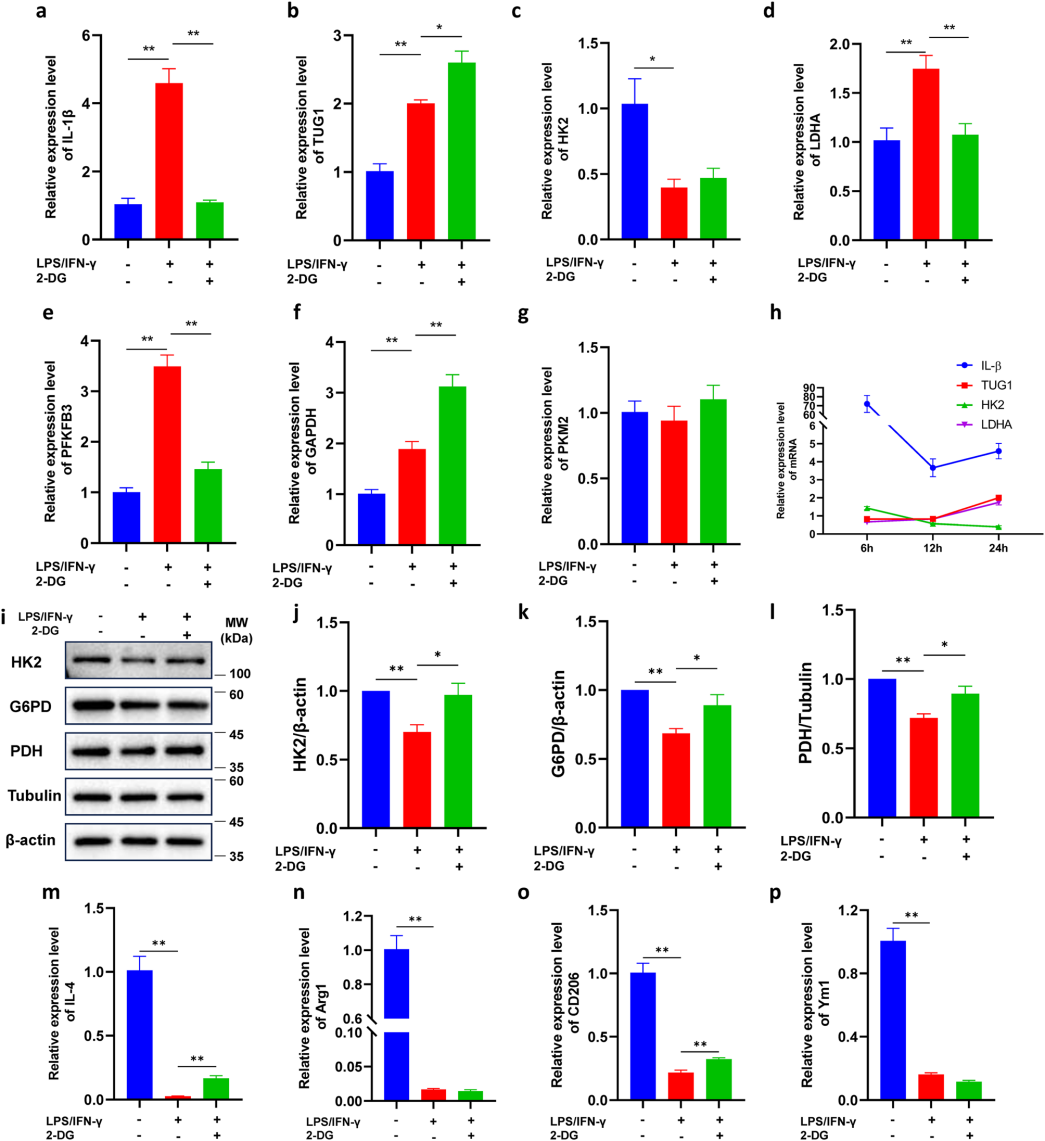
TUG1 is involved in the regulation of glucose metabolism in microglial activation. (**a**–**g**) The expression of IL-1β,TUG1, HK2, LDHA, PFKFB3, GAPDH, and PKM2 mRNA after induced with LPS (100 ng/ml)/IFN-γ(10 ng/ml) and with or without 2-DG for 24 h (n = 3). (**h**) Time curve of relative expression levels of IL-1β, TUG1, HK2, and LDHA mRNA. (**i**) Western blot was conducted to determine the expression of the key enzymes HK2, G6PD, and PDH after induced with LPS (100 ng/ml)/IFN-γ(10 ng/ml) and with or without 2-DG for 24 h. The immunoblot image represents three independent experiments with similar results. (**j**) Statistical analysis of data for the expression of HK2 (n = 3). (**k**) Statistical analysis of data for the expression of G6PD (n = 3). (**l**) Statistical analysis of data for the expression of PDH (n = 3). (**m**–**p**) The expression of IL-4, Arg1, CD206, and Ym1 mRNA after induced with LPS (100 ng/ml)/IFN-γ (10 ng/ml) and with or without 2-DG for 24 h (n = 3). Error bars indicate SEM. *P < 0.05, **P < 0.01.

To further explore the role of TUG1 and glucose metabolism in microglial activation, we detected the expression levels of key enzymes of the glycolytic pathway. Compared to the control group, the expression of IL-1β mRNA was up-regulated at any time we detected. Meanwhile, the intervention of 2-deoxy-D-glucose (2-DG), a glycolysis inhibitor, can reduce the expression of inflammatory factors (Figs. 3a, S2a, e). The expression of anti-inflammatory cytokines IL-4, Arginase 1(Arg-1), CD206, and Ym1 were decreased when induced by LPS/IFN-γ, while this decrease was reversed by the treatment with 2-DG (Fig. 3m-p). The expression of TUG1, Lactate dehydrogenase A(LDHA), 6-Phosphofructo-2-kinase/fructose-2,6-bisphosphatase isoform 3 (PFKFB3), and GAPDH mRNA was also up-regulated, while the expression of Pyruvate kinase isozyme typeM2 (PKM2) did not significantly change (Fig. 3b, d-f). The expression of hexokinase2 (HK2) mRNA was up-regulated at 6 h and downregulated after 12 h (Figure S2g, c), while the expression of LDHA and TUG1 mRNA had the opposite result to HK2 (Figs. 3b-d, S2f, b, h, d). The expression of IL-β, TUG1, HK2, and LDHA exhibit temporal variability (Fig. 3h). The protein levels of HK2, G6PD, and PDH were significantly higher than the normal control group, while 2-DG reversed this upregulation at 6 h (Figure S2i-l). However, the protein levels of HK2, G6PD, and PDH were significantly lower than the normal control group at 24 h (Fig. 3i-l). Those results suggested that the elevated TUG1 expression levels promote sustained proinflammatory activation in microglia.

### TUG1 knockout altered the expression of key enzymes in the glycolytic pathway in microglia

**Figure 4:**
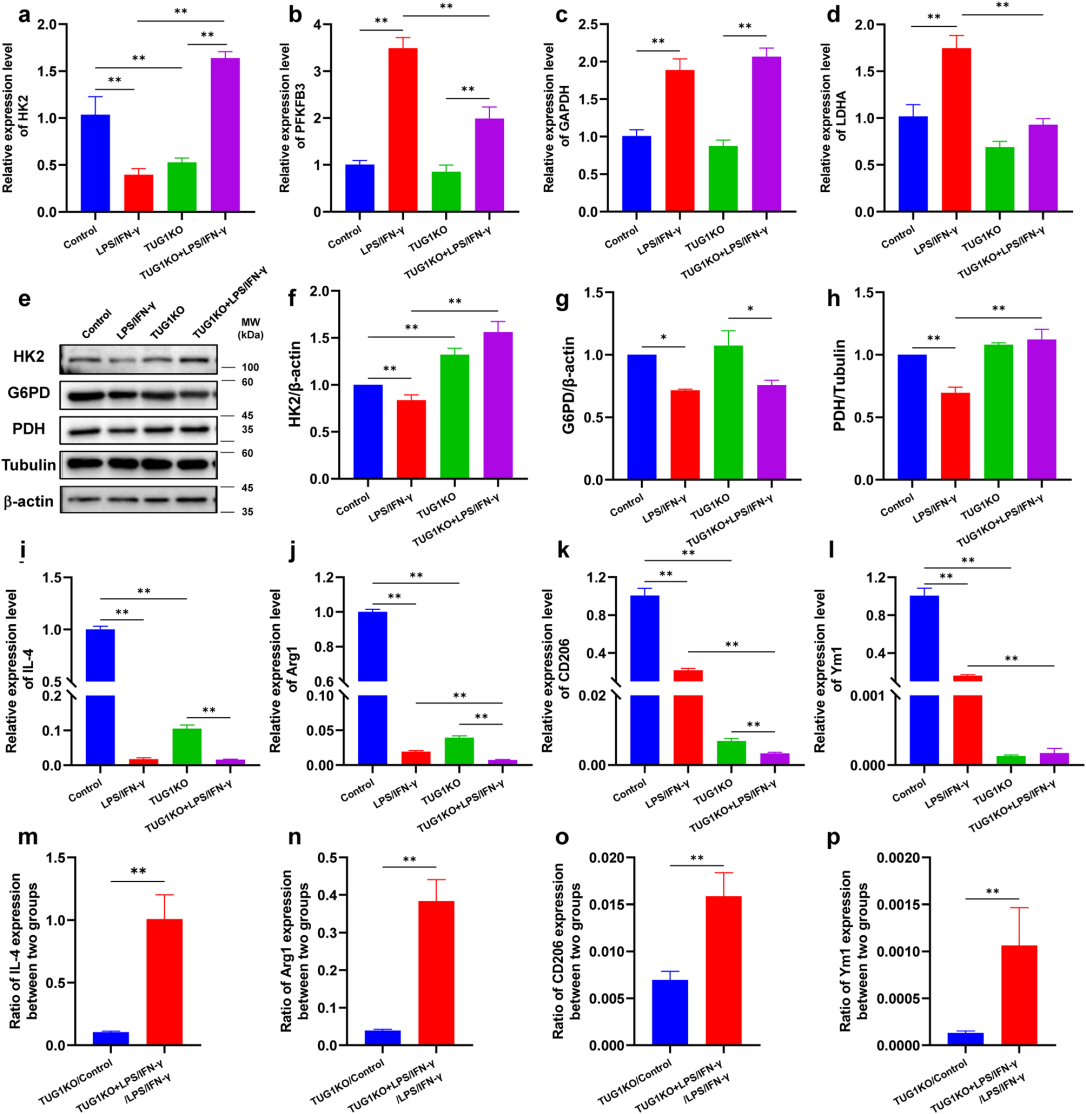
TUG1 knockout altered the expression levels of key enzymes in the glycolytic pathway in microglia. (**a**–**d**) The expression of HK2, PFKFB3, GAPDH, and LDHA mRNA after induced with LPS (100 ng/ml)/IFN-γ(10 ng/ml) for 24 h (n = 3). (**e**) Western blot was conducted to determine the expression of the key enzymes HK2, G6PD, and PDH after induced with LPS (100 ng/ml)/IFN-γ(10 ng/ml) for 24 h. The immunoblot image represents three independent experiments with similar results. (**f**) Statistical analysis of data for the expression of HK2 (n = 3). (**g**) Statistical analysis of data for the expression of G6PD (n = 3). (**h**) Statistical analysis of data for the expression of PDH (n = 3). (**i**–**j**) The expression of IL-4, Arg1, CD206, and Ym1 mRNA after induced with LPS (100 ng/ml)/IFN-γ(10 ng/ml) for 24 h (n = 3). (**m**–**p**). The ratio of IL-4, Arg1, CD206, and Ym1 expression levels in the TUG1KO group compared to the control group, and the TUG1KO induced with LPS/IFN-γ group compared to the LPS/IFN-γ group (n = 3). Error bars indicate SEM. *P < 0.05, **P < 0.01.

To further demonstrate the role of TUG1 in modulating glucose metabolism during inflammatory activation of BV2 cells, we constructed TUG1 knockout (TUG1KO) BV2 by CRISPR-Cas9 (Figure S1) and performed PCR analysis on key enzymes involved in the glycolytic pathway. The results showed that compared to the control group, the mRNA expression levels of HK2 were significantly decreased (Fig. 4a), while the mRNA expression levels of PFKFB3, GAPDH, and LDHA were significantly increased in BV2 cells under inflammatory activation (Fig. 4b-d). This suggests that inflammatory activation can upregulate the mRNA expression levels of key enzymes to enhance glycolysis. TUG1 knockout decreased the expression of HK2 but did not significantly change the expression of PFKFB3, GAPDH, and LDHA (Fig. 4a-d). In comparison to the LPS/IFN-γ group, TUG1KO induced with LPS/IFN-γ resulted in a significant decrease in mRNA expression levels of PFKFB3 and LDHA, along with an increase in HK2 mRNA expression levels (Fig. 4a-b, 4d). The induction of both LPS/IFN-γ and TUG1KO led to a decrease in the expression of IL-4, Arg1, CD206, and Ym1 mRNA (Fig. 4i-l). Nevertheless, upon comparing the ratios of TUG1KO to control and TUG1KO induced with LPS/IFN-γ to that of LPS/IFN-γ alone, it became evident that TUG1KO had a restorative effect, and could potentially confer benefits to microglia while also shielding them from proinflammatory activation when induced with LPS/IFN-γ (Fig. 4m-p). This indicates that TUG1 knockout can reverse the changes in key enzymes involved in glycolysis in proinflammatory-activated BV2 cells. These findings suggest a potential role for TUG1 in regulating glycolysis in proinflammatory-activated BV2 cells through the modulation of these enzymes.

To further elucidate the influence of TUG1 on glucose metabolism, we investigated the expression levels of key enzymes involved in the three major pathways of glucose metabolism. Our findings revealed a significant decrease in the protein expression levels of HK2, G6PD, and PDH in the LPS/IFN-γ group compared to the control group (Fig. 4e-h). When comparing the TUG1KO group to the control group, the expression of HK2 was increased, while the expression of G6PD and PDH did not observe any significant changes. When comparing the TUG1KO induced with the LPS/IFN-γ group to the LPS/IFN-γ group, we did not observe any significant changes in G6PD protein expression levels (Fig. 4g). However, there was a notable increase in the protein expression levels of HK2 and PDH (Fig. 4f, h). These results suggest that TUG1KO partly counteracts the attenuated oxidative phosphorylation observed in proinflammatory-activated microglia. Furthermore, we discovered that inhibiting the glycolysis pathway to a certain extent promotes compensatory enhancement in the pentose phosphate pathway and that TUG1KO strengthens this compensatory mechanism. In summary, our study provides compelling evidence that TUG1 knockout alters the protein levels of key enzymes in the glycolytic pathway in BV2 cells under proinflammatory activation. These findings expand our understanding of the regulatory role of TUG1 in glucose metabolism and shed light on potential therapeutic targets for inflammatory conditions.

### TUG1 knockout reverses the enhanced glycolysis rate induced by LPS/IFN-γ in microglia

**Figure 5:**
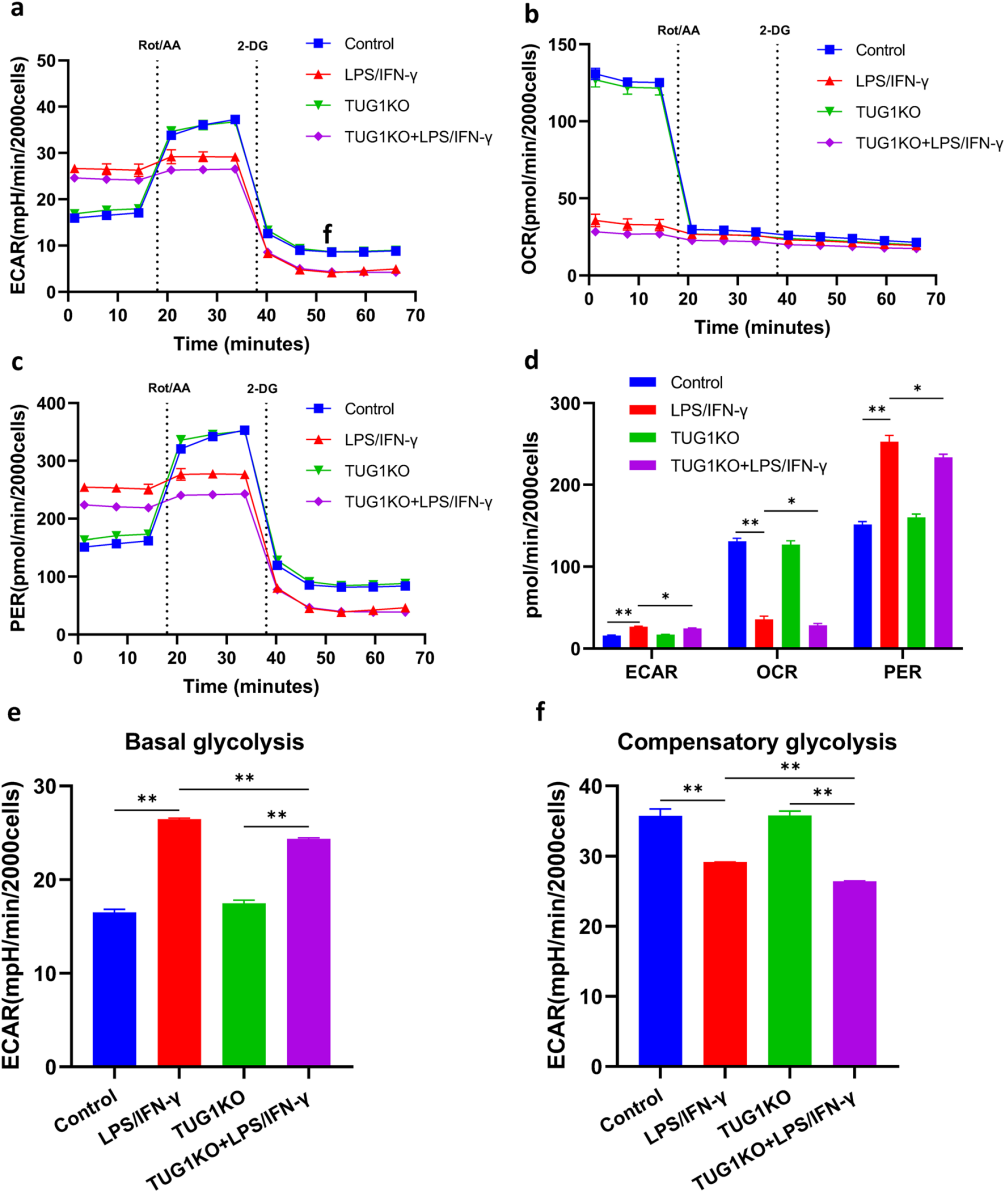
TUG1-KO reverses the enhanced glycolysis rate induced by LPS/IFN-γ in microglia. (**a**) Real-time changes in the extracellular acidification rate (ECAR) were measured using Seahorse XF. The cells were sequentially treated with Rot/AA and 2-DG (n = 3 biologically independent samples). (**b**) Real-time changes in the oxygen consumption rate (OCR) were measured using Seahorse XF. The cells were sequentially treated with Rot/AA and 2-DG (n = 3 biologically independent samples). (**c**) Real-time change in the proton efflux rate (PER). The cells were sequentially treated with Rot/AA and 2-DG (n = 3 biologically independent samples). (**d**) Statistical analysis of data for ECAR, OCR, and PER (n = 3). (**e**) Statistical analysis of data for basal glycolysis (n = 3). (**f**) Statistical analysis of data for compensatory glycolysis (n = 3). Error bars indicate SEM. *P < 0.05, **P < 0.01.

To further confirm whether TUG1 regulates the metabolic activity of microglia, we assessed the rate of glycolysis by measuring OCR, ECAR, and PER in BV2 cells using Glycolytic Rate Assay. we performed experiments using inhibitors such as Rotenone/Antimycin A (Rot/AA) and 2-Deoxy-D-Glucose (2-DG) to selectively inhibit aerobic respiration and glycolysis, respectively. Compared to the control group, LPS/IFN-γ intervention leads to an increase in glycolysis levels and a decrease in oxidative phosphorylation levels, thereby increasing the proton efflux rate of glycolysis. TUG1KO did not change both glycolysis and oxidative phosphorylation levels. After administering LPS/IFN-γ intervention, TUG1KO can decrease extracellular acidification rates and the proton efflux rate of glycolysis, indicating an increase in mitochondrial acidification and simultaneous elevation of oxidative phosphorylation levels (Fig. 5a-d). The induction of LPS/IFN-γ was found to increase the basal glycolysis and decrease the compensatory glycolysis of BV2 cells. However, in TUG1KO BV2 cells, the basal and compensatory glycolysis were not affected under basal conditions. When induced with LPS/IFN-γ, TUG1KO cells showed a significant reduction in both basal and compensatory glycolysis, compared to the LPS/IFN-γ group (Fig. 5e-f). These results suggest that TUG1KO can to some extent sustain normal energy supply processes and inhibit glycolysis, thus avoiding excessive activation of microglial cells.

### TUG1 knockout alleviates the accumulation of metabolites and the blocked TCA cycle in microglia

**Figure 6:**
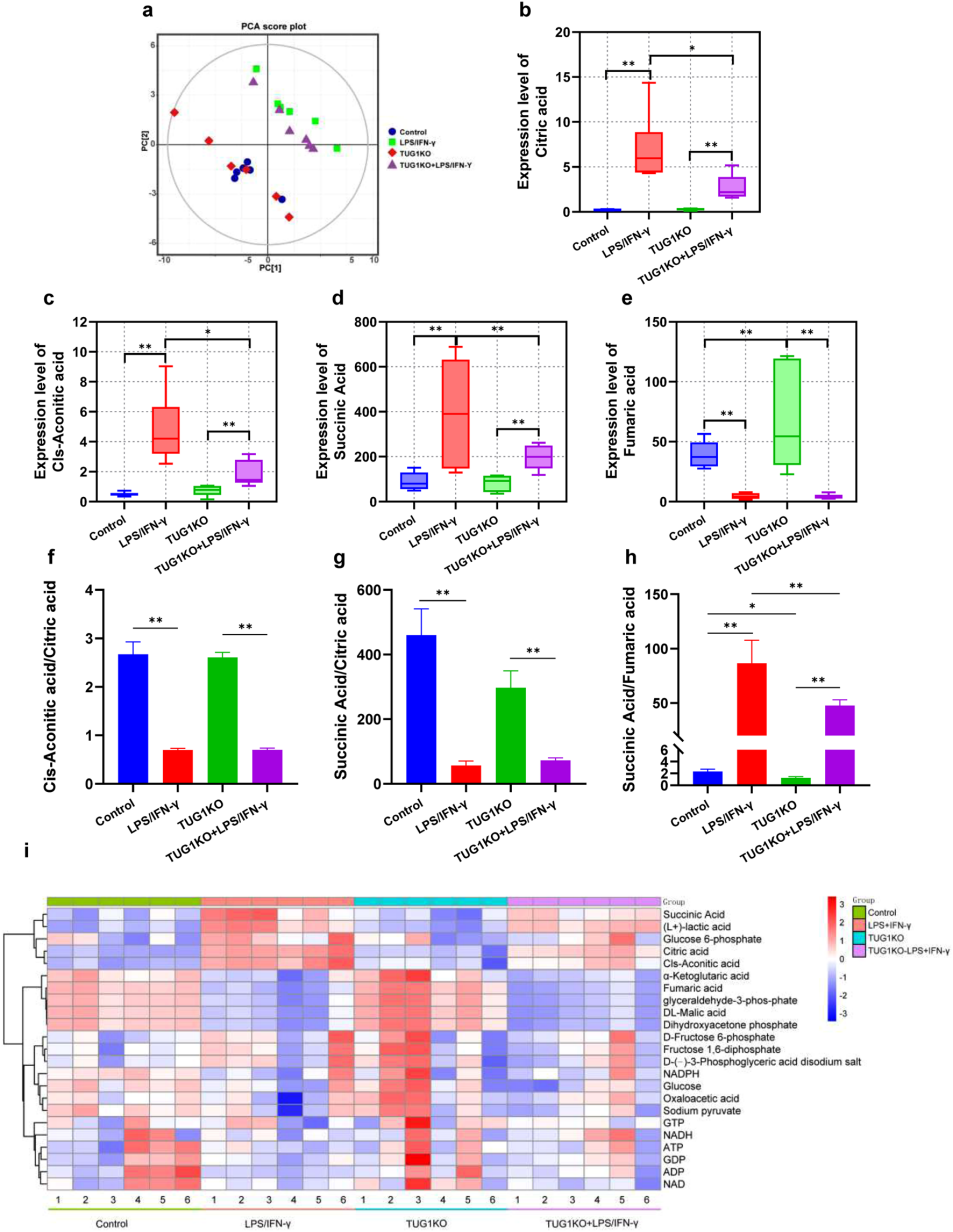
TUG1 knockout alleviates the accumulation of metabolism and the blocked TCA cycle in microglia. (**a**) The Principal Component Analysis (PCA) of groups (n = 6). (**b**) The expression level of differential metabolites Citric acid of different groups (n = 6). (**c**) The expression level of differential metabolites Cis-Aconitic acid of different groups (n = 6). (**d**) The expression level of differential metabolites Succinic acid of different groups (n = 6). (**e**) The expression level of differential metabolites Fumaric acid of different groups (n = 6). (**f**) The ratio of Cis-Aconitic acid to Citric acid. (**g**) The ratio of Succinic acid to Citric acid. (**h**) The ratio of Succinic acid to Fumaric acid. (**i**) Heatmap of differential metabolites between the different groups (n = 6). Error bars indicate SEM. *P < 0.05, **P < 0.01.

To further investigate the regulatory role of TUG1 in glucose metabolism, we performed a metabolomics analysis. Principal component analysis (PCA) revealed distinct clustering patterns among the groups (Fig. 6a). Compared to the control group, the induction of LPS/IFN-γ exhibited upregulation of Citric acid, Cis-Aconitic acid, and Succinic acid, while the expression levels of metabolites such as Fumaric acid and DL-Malic acid were downregulated, and the TUG1KO group showed an increase in the expression level of Fumaric acid (Fig. 6b-e, i). The ratio of Cis-Aconitic acid and Succinic acid to Citric acid was both lower, while the ratio of Succinic acid to Fumaric acid was higher (Fig. 6f-h). In comparison to the LPS/IFN-γ group, the TUG1KO induced with LPS/IFN-γ showed a decrease in the expression levels of Citric acid, Cis-Aconitic acid, and Succinic acid (Fig. 6b-d, i), and the ratio of Succinic acid to Fumaric acid was decreased (Fig. 6e, i). These results suggest that TUG1 knockout alleviates the accumulation of metabolites and the blocked TCA cycle in proinflammatory-activated BV2 cells.

### The TCA cycle is a crucial pathway regulated by TUG1 in the activation of microglia

**Figure 7:**
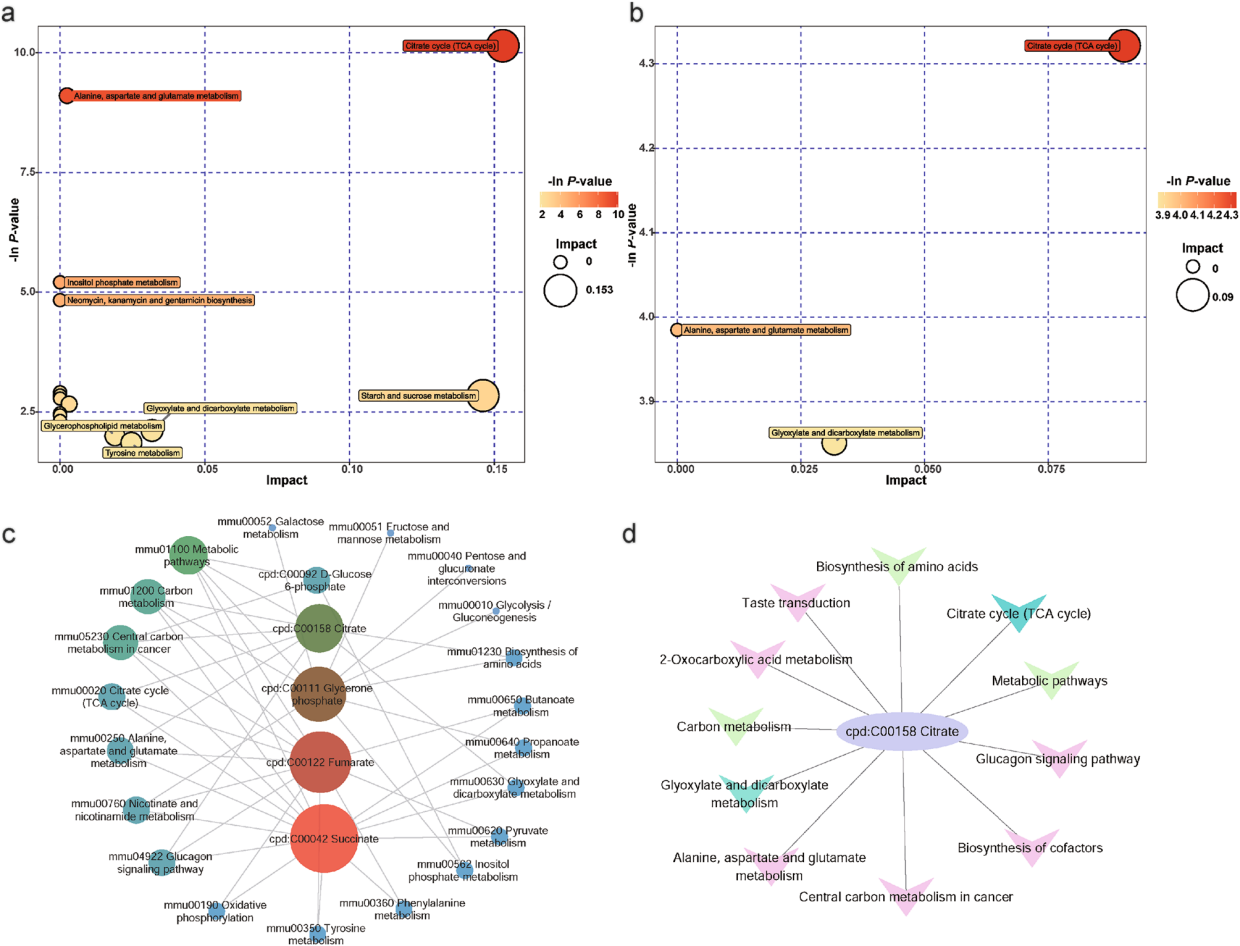
The TCA cycle is a crucial pathway regulated by TUG1 in the activation of microglia. (**a**) Bubble plot of pathway enrichment of differential metabolites between the control and LPS/IFN-γ group. (**b**) Bubble plot of pathway enrichment of differential metabolites between the LPS/IFN-γ group and TUG1KO induced with LPS/IFN-γ group. (**c**) Pathway enrichment plot of differential metabolites between the control and LPS/IFN-γ group. (**d**) Pathway enrichment plot of differential metabolites between the LPS/IFN-γ group and the TUG1KO induced with LPS/IFN-γ group.

To further elucidate the involvement of TUG1 in the regulation of glucose metabolism, we performed pathway enrichment analysis on differentially expressed metabolites. The results revealed a high enrichment rate of the TCA cycle pathway in both the LPS/IFN-γ and TUG1KO induced with LPS/IFN-γ groups compared to the control group (Fig. 7a-d). This finding suggests that the TCA cycle may be a crucial pathway in which TUG1 participates in the regulation of glucose metabolism. Furthermore, considering the expression levels of metabolites, TUG1 knockout significantly reverses the inhibition of the TCA cycle under inflammatory conditions. This not only provides energy but also avoids excessive activation of microglial cells and enhancement of inflammation that may result from increased glycolysis.

## Discussion

Inflammatory activation of microglial cells, coupled with a switch from oxidative phosphorylation to glycolysis, is a key characteristic of Alzheimer's disease (AD)^14^. The glycolysis/H4K12la/PKM2 positive feedback loop worsens the functional impairment of microglial cells in AD. Intriguingly, inhibiting PKM2 with pharmacological agents eases microglial cell activation, while selectively removing PKM2 in microglia enhances spatial learning and memory abilities in AD mice^16^. Furthermore, hexokinase 2 (HK2), an important glycolytic enzyme, influences microglial-mediated neuroinflammation by promoting the transcription and expression of IL-1β^17^. Neuronal death in neurodegenerative diseases like AD and Parkinson's disease (PD) is associated with the nuclear translocation, aggregation, and binding of glyceraldehyde-3-phosphate dehydrogenase (GAPDH) to toxic proteins such as β-amyloid peptides, tau protein, and α-synuclein^18^. TUG1 regulates microglial activation by sequestering miR-145a-5p. TUG1 contributes to NLRP3 inflammasome-dependent pyroptosis through the miR-145a-5p/TLR4 axis following stroke, leading to the release of inflammatory mediators and resulting in inflammation-related damage^19^. Interestingly, TUG1 has also been linked to glycolysis. In the context of cancer, exosomal TUG1 derived from cancer-associated fibroblasts promotes liver cancer cell migration, invasion, and glycolysis through modulation of the miR-524-5p/SIX1 axis^12^. Additionally, TUG1 knockdown enhances the cytotoxic effects of adriamycin in HL60/ADR cells by inhibiting glycolysis through the inactivation of the Akt pathway^20^. Given these intriguing findings, it is imperative to investigate the intricate relationship between TUG1, inflammation, and glucose metabolism in microglia. Such studies hold promise for identifying potential therapeutic targets to combat neuroinflammation and neurodegenerative diseases like AD and PD.

### Principal findings and comparison with other studies

Our study revealed a noteworthy finding regarding the upregulation of TUG1 in pro-inflammatorily activated microglial cells, showing a positive correlation with the expression of inflammatory cytokines. Surprisingly, we observed that silencing of GAPDH led to an increase in TUG1 expression and elevated levels of inflammatory cytokines. This contradictory result challenges previous findings suggesting that GAPDH nuclear translocation overactivates microglial cells under oxidative stress conditions, exacerbating injury^21^. We propose that the conflicting observations could be attributed to the upregulation of TUG1 expression caused by GAPDH silencing, thereby facilitating an enhanced inflammatory response in microglial cells. Meanwhile, GAPDH is the glycolytic enzyme in mice and humans, GAPDH inhibition downregulates aerobic glycolysis in myeloid and lymphoid cells, preventing immune activation and shifting the balance between inflammatory and regulatory cell types^15^. Additionally, during development, growth, and disease, microglia display various phenotypes. Recent studies have proposed that these phenotype transitions are orchestrated by the reprogramming of cellular metabolism. It is evident that metabolic pathways undergo distinct alterations in activated microglia and act as critical regulators of microglial responses^22^. Previous studies have shown that proinflammatory microglia are powered by glycolysis, which relies on high levels of glucose uptake^23^. Based on our experimental results, we speculate that TUG1 may be involved in microglial proinflammatory activation through its participation in glucose metabolism, considering its impact on the expression of GAPDH. Therefore, we have designed follow-up experiments to investigate the regulation of glycolysis by TUG1 in microglial proinflammatory activation.

We observed a significant increase in glycolysis and a decrease in oxidative phosphorylation in inflammatorily activated microglial cells, leading to an accelerated glycolytic proton efflux rate. This finding aligns with previous studies demonstrating enhanced glycolytic metabolism and reduced mitochondrial activity in inflammatory M1 macrophages^24^. Interestingly, our study revealed no significant differences between the normal control group and the TUG1 knockout group under normal conditions. However, when exposed to LPS/IFN-γ stimulation, the TUG1 knockout group exhibited a noticeable decrease in the extracellular acidification rate (ECAR) and the proton efflux rate (PER), which collectively represent total extracellular acidification^25^. Total extracellular acidification consists of two components: respiratory acidification from CO_2_ production in the citric acid cycle and anaerobic glycolytic acidification from lactate production^26^. These findings suggest that TUG1 knockout does not affect microglial energy production under normal conditions but inhibits the augmented glycolysis seen in inflammatorily activated microglia. Therefore, our results indicate that TUG1 plays a regulatory role in glycolytic metabolism in microglia during inflammation.

Our study demonstrated that activated microglia exhibit heightened glycolysis, and the knockout of TUG1 can effectively inhibit glycolysis in these cells. This inhibition leads to a shift in glucose metabolism towards oxidative phosphorylation and the pentose phosphate pathway. Prior research has established that glycolysis is the primary energy source for inflammatory activation of microglial cells, whereas oxidative phosphorylation is the dominant energy source for anti-inflammatory activation^6^. By promoting this metabolic transition from glycolysis to oxidative phosphorylation, the downregulation of TUG1 facilitates the conversion of proinflammatory-activated microglia into an anti-inflammatory phenotype. This metabolic shift holds great potential for improving brain inflammation and exerting protective effects. The pentose phosphate pathway, which generates NADPH, can effectively suppress oxidative stress and neuroinflammation. Furthermore, exogenous administration of NADPH has been found to inhibit oxidative stress and modulate multiple signaling pathways, thereby suppressing neuroinflammation. However, it is important to note that NADPH oxidases (NOX), which utilize NADPH as a substrate, can generate reactive oxygen species (ROS) and contribute to neuroinflammation^27^. Therefore, the enhancement of the pentose phosphate pathway and the increase in NADPH levels can, to some extent, promote antioxidant responses and maintain a reducing intracellular environment, thereby protecting cells from oxidative damage induced by inflammation.

Furthermore, our findings suggest that TUG1 plays a significant role in the regulation of glucose metabolism and is associated with the tricarboxylic acid (TCA) cycle, which is essential for oxidative phosphorylation. Previous studies have indicated that M1 macrophages primarily rely on glycolysis and exhibit disruptions in the TCA cycle, resulting in the accumulation of itaconate (a microbicidal compound) and succinate^6^. Through metabolomics analysis, we observed increased levels of citric acid, cis-aconitic acid, and succinic acid, as well as decreased expression of fumaric acid in inflamed microglia. Moreover, the ratios of cis-aconitic acid to citric acid and succinic acid to citric acid showed significant decreases, while the succinic acid to fumaric acid ratio exhibited a significant increase. However, in activated microglia lacking TUG1, the succinic acid to fumaric acid ratio decreased. The accumulation of cis-aconitic acid and succinic acid, which are intermediate metabolites of the TCA cycle, suggests a blockade in oxidative phosphorylation^28^. Notably, LPS strongly increases succinate levels, which serves as an inflammatory signal that enhances interleukin-1β production during inflammation^29^. Hence, the knockout of TUG1 appears to improve the blockade in the TCA cycle and inflammation in microglia.

Furthermore, we observed increased expression of lactate dehydrogenase A (LDHA) and subsequent lactate generation in inflamed microglia, indicating enhanced glycolysis. LDHA activity is vital for the proinflammatory phenotype of macrophages^30^. TUG1 may play a crucial role in maintaining LDHA-dependent lactate production in inflammatory microglia. Additionally, the expression of pyruvate dehydrogenase (PDH) was decreased, impairing the complete oxidation of pyruvate via the TCA cycle. These results are consistent with previous studies that demonstrate a metabolic shift in microglia from oxidative phosphorylation to aerobic glycolysis under inflammatory conditions^31^. However, the knockout of TUG1 appeared to ameliorate this condition, reducing the accumulation of citric acid and cis-aconitic acid and promoting the restoration of the TCA cycle. This restoration, in turn, enhanced oxidative phosphorylation and mitigated the compensatory increase in glycolysis. As a result, TUG1 knockout improved energy production and alleviated inflammation activation in microglia. Thus, our findings support the notion that the TCA cycle may represent a crucial pathway through which TUG1 participates in the regulation of glucose metabolism.

### Strengths and limitations of the study

Our study has unveiled the significant involvement of TUG1 in modulating glucose metabolism and orchestrating microglial activation and inflammation. This comprehensive investigation not only enriches the body of knowledge about lncRNA in disease regulation but also provides a fresh perspective for the exploration of chronic inflammatory disorders, such as neuroinflammation. Neuroinflammation is instigated by the misfiring of immune cells in the CNS involving microglia and astrocytes as key cell types. It is a favorable as well as a detrimental process for neurodevelopment and associated processes. The chronic or uncontrolled inflammatory responses may lead to various neurodegenerative diseases, including Alzheimer's disease (AD), Parkinson's disease (PD), amyotrophic lateral sclerosis, and multiple sclerosis^32^.

Although there are important discoveries revealed by these studies, there are also limitations. Firstly, we have only conducted in vitro experiments to study the regulation of TUG1 on glucose metabolism in activated microglia. Further research is needed to explore the role of TUG1 in vivo glucose metabolism and regulation of inflammation. Additionally, since changes in glucose metabolism and inflammation can mutually influence each other, the specific targets of TUG1 need to be further clarified. Some of the results showed no significant differences between the control group and the TUG1KO group, but differences were observed under LPS/IFN-γ intervention. This suggests that TUG1 may have multiple targets in regulating glucose metabolism and activation of microglia, and its effects could be influenced by the intracellular environment. The specific mechanism warrants further investigation.

## Conclusion

The present study suggests that TUG1 is involved in regulating glucose metabolism in activated microglia, leading to the accumulation of metabolites such as Citric acid and Cis-Aconitic acid through the inhibition of the TCA cycle. Downregulation of TUG1 inhibits glycolysis and restores the blocked TCA cycle, promoting a shift in microglial glucose metabolism from glycolysis to oxidative phosphorylation. This metabolic shift is accompanied by a transition from pro-inflammatory activation to anti-inflammatory activation in microglia, thereby exerting an anti-inflammatory effect.

### Supplementary Information

Supplementary Information 1.

Supplementary Information 2.

Supplementary Information 3.

Supplementary Information 4.

Supplementary Information 5.

## Data Availability

Data is provided within the manuscript or supplementary information files.
