# Supplementary figures and images for: LncRNA TUG1 mediates microglial inflammatory activation by regulating glucose metabolic reprogramming
Source: Sci Rep. 2024 May 27;14:12143. doi: 10.1038/s41598-024-62966-4 (PMC11130314; doi:10.1038/s41598-024-62966-4)

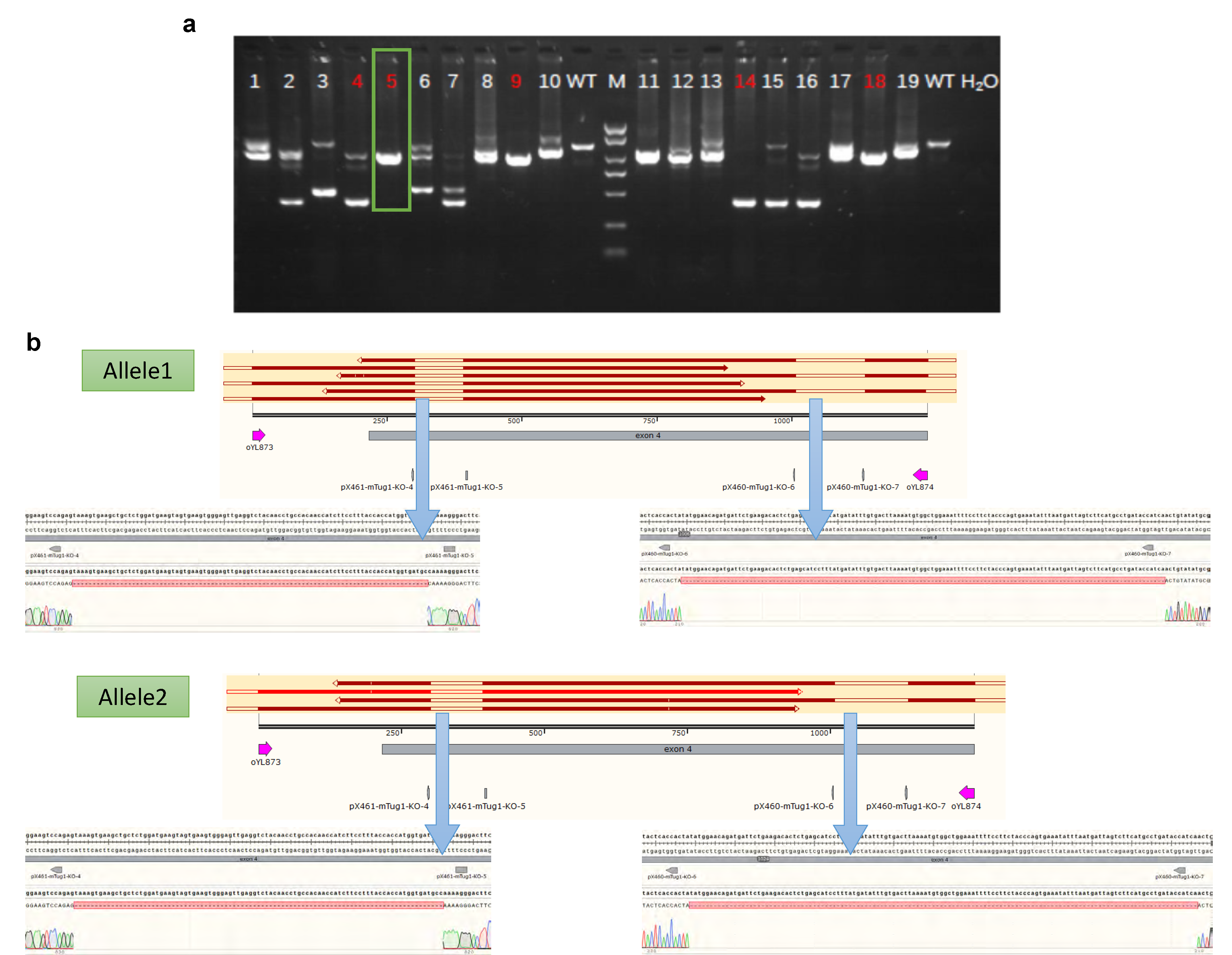

Supplement: Supplementary file 1 — Supplementary Information 1. [file 41598_2024_62966_MOESM1_ESM.tif]

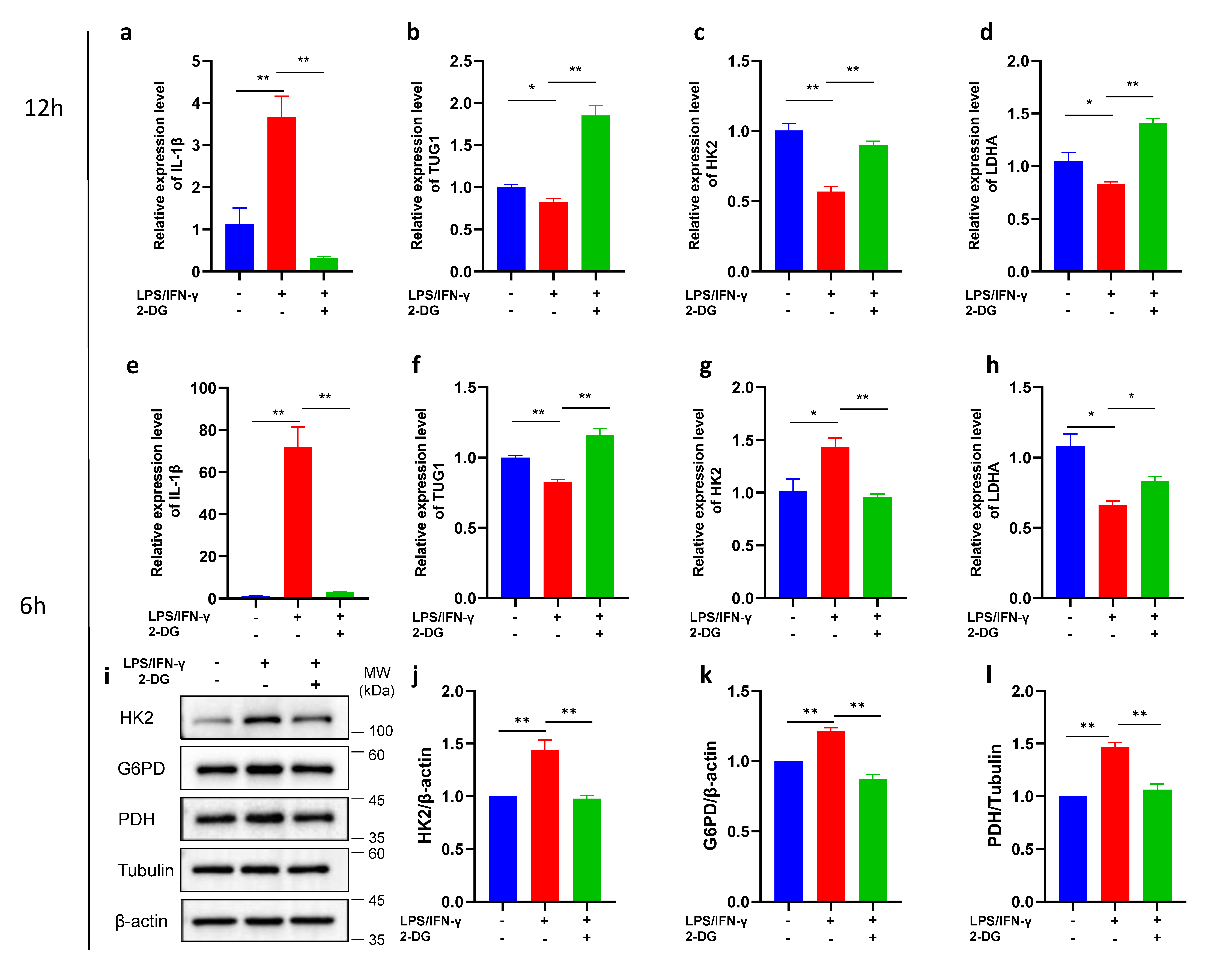

Supplement: Supplementary file 2 — Supplementary Information 2. [file 41598_2024_62966_MOESM2_ESM.tiff]
