# Supplementary material for: LncRNA TUG1 mediates microglial inflammatory activation by regulating glucose metabolic reprogramming
Source: Sci Rep. 2024 May 27;14:12143. doi: 10.1038/s41598-024-62966-4 (PMC11130314; doi:10.1038/s41598-024-62966-4)
Supplement: Supplementary file 3 — Supplementary Information 3. [file 41598_2024_62966_MOESM3_ESM.docx]

**Supplementary materials**

**Table S1. sgRNA sequence**

**Figure S1 Agarose gel electrophoresis results for monoclonal screening of TUG1 KO.**

(a).M: DNA Marker I (700, 600, 500, 400, 300, 200, 100 bp). WT: BV2 wild-type genomic DNA control.

H_2_O: negative control. NO. 1-19: the different monoclonal TUG1 KO BV2. NO.5 was selected for the total research. (b). Sanger DNA sequencing of the knockout cell line.

**Figure S2 TUG1 is involved in the regulation of glucose metabolism in microglial activation.**

(a-d). The expression of IL-1β, TUG1, HK2, and LDHA mRNA after induced with LPS (100ng/ml)/IFN-γ(10ng/ml) and with or without 2-DG for 12h (n = 3). (e-h). The expression of IL-1β, TUG1, HK2, and LDHA mRNA after induced with LPS (100ng/ml)/IFN-γ(10ng/ml) and with or without 2-DG for 6h (n = 3). (i). Western blot was conducted to determine the expression of the key enzymes HK2, G6PD, and PDH after induced with LPS (100ng/ml)/IFN-γ(10ng/ml) and with or without 2-DG for 6h. The immunoblot image represents three independent experiments with similar results. (j). Statistical analysis of data for the expression of HK2 (n = 3). (k). Statistical analysis of data for the expression of G6PD (n = 3). (l). Statistical analysis of data for the expression of PDH (n = 3). Error bars indicate SEM. **P* < 0.05, ***P* < 0.01.
