# Supplementary material for: LncRNA TUG1 mediates microglial inflammatory activation by regulating glucose metabolic reprogramming
Source: Sci Rep. 2024 May 27;14:12143. doi: 10.1038/s41598-024-62966-4 (PMC11130314; doi:10.1038/s41598-024-62966-4)
Supplement: Supplementary file 4 — Supplementary Information 4. [file 41598_2024_62966_MOESM4_ESM.docx]

**Supplementary Table 1.** **sgRNA sequence**

| **sgRNA** | **sequence** |
| --- | --- |
| KO-1 | TGTCTTCTTCAGTAGTAAGG TGG |
| KO-2 | GACTCTTGACTGCACTATAA AGG |
| KO-3 | GTATCATCTTCGGGTTACTC AGG |
| KO-4 | AGAGCAGCTTCACTTTACTC TGG |
| KO-5 | ACCACCATGGTGATGCCAAA AGG |
| KO-6 | GAATCATCTGTTCCATATAG TGG |
| KO-7 | TATCACGCATATACAGTTGA TGG |
