# Supplementary material for: LncRNA TUG1 mediates microglial inflammatory activation by regulating glucose metabolic reprogramming
Source: Sci Rep. 2024 May 27;14:12143. doi: 10.1038/s41598-024-62966-4 (PMC11130314; doi:10.1038/s41598-024-62966-4)

Figure 3i Western blot

HK2(102kDa)

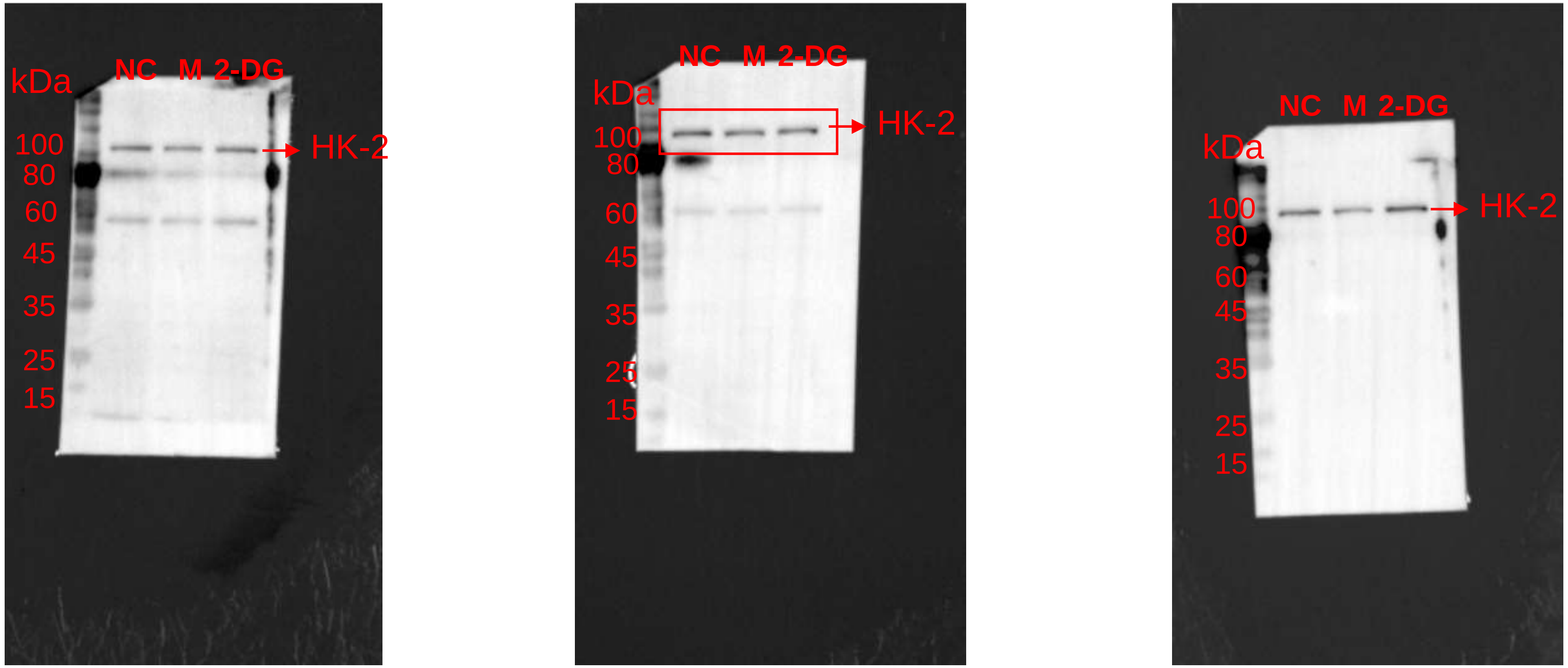

Figure 3i Western blot

G6PD(59kDa)

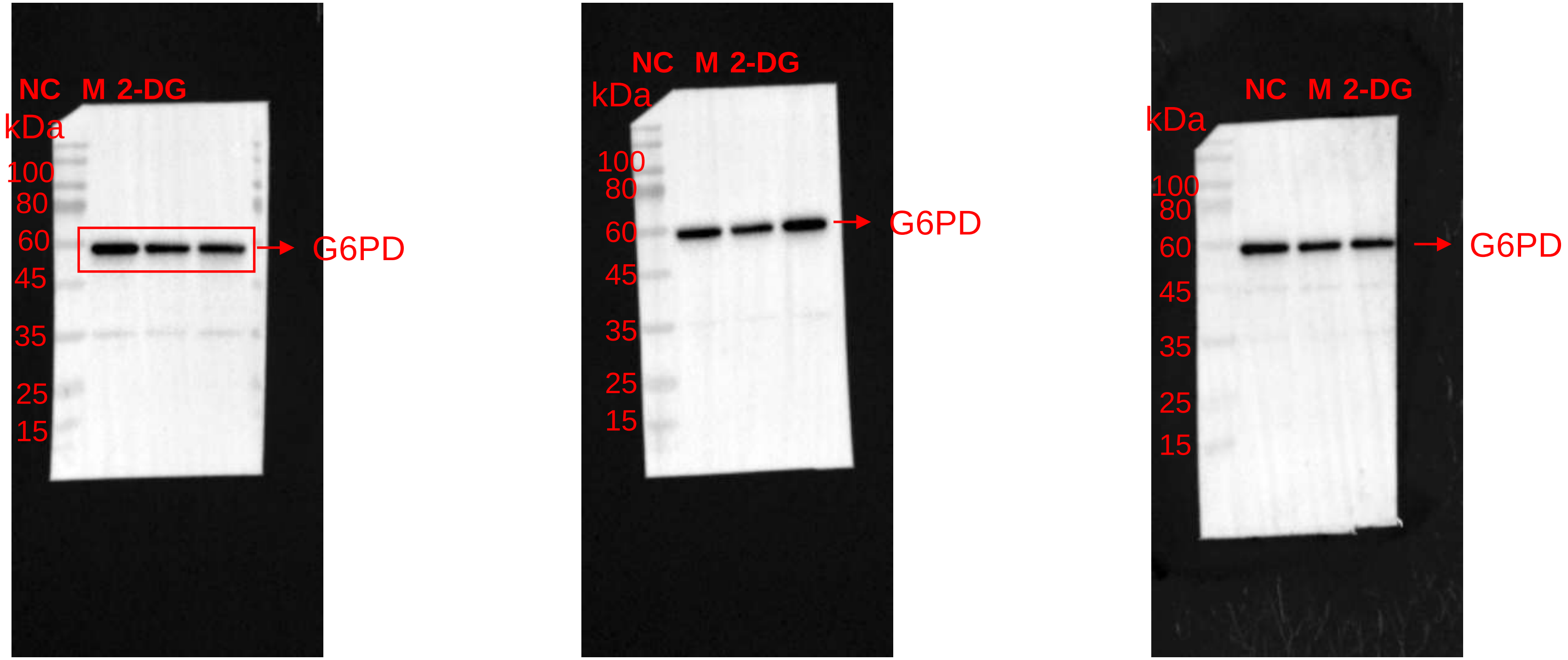

Figure 3i Western blot

PDH(43kDa)

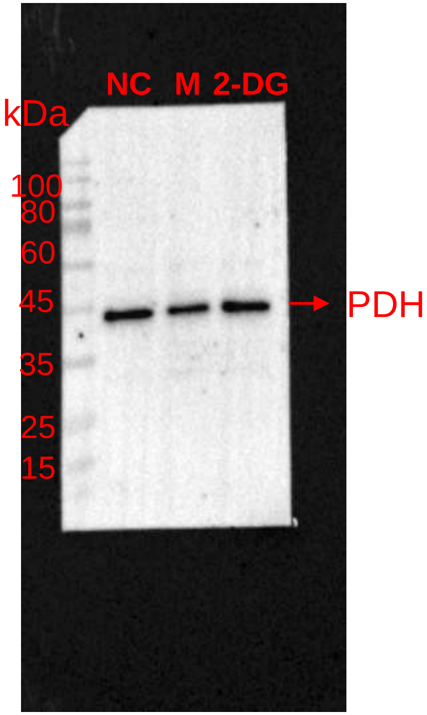

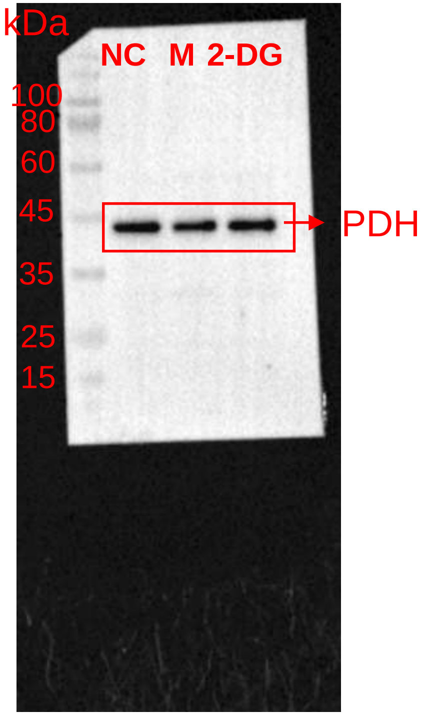

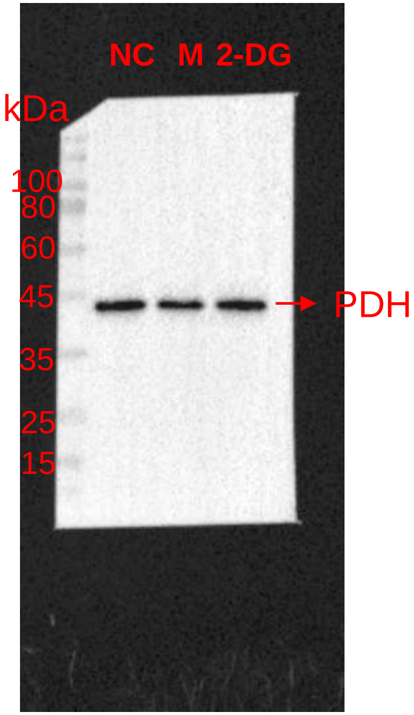

Figure 3i Western blot

Tubulin(55kDa)

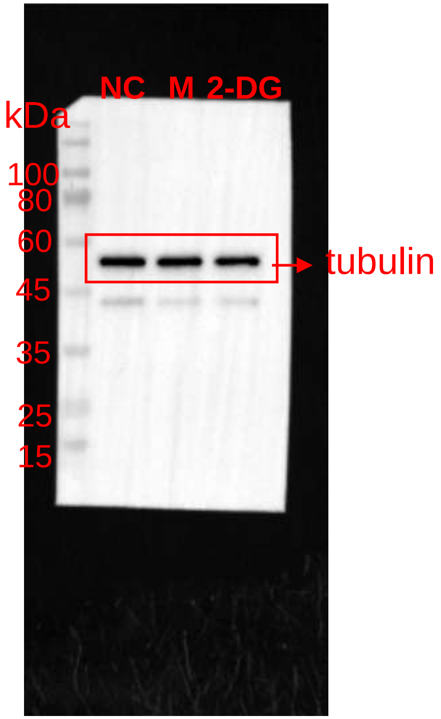

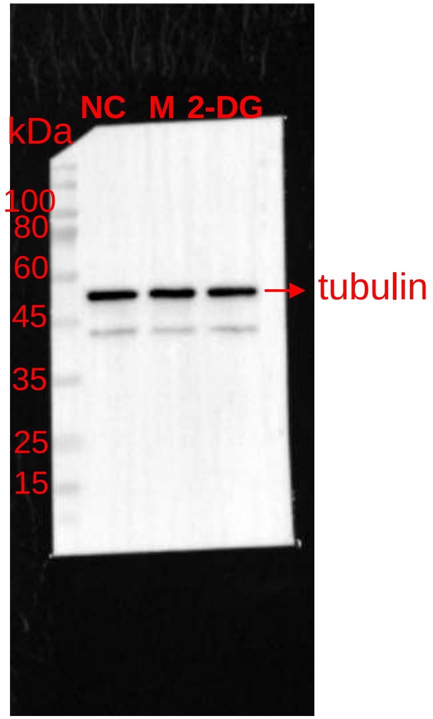

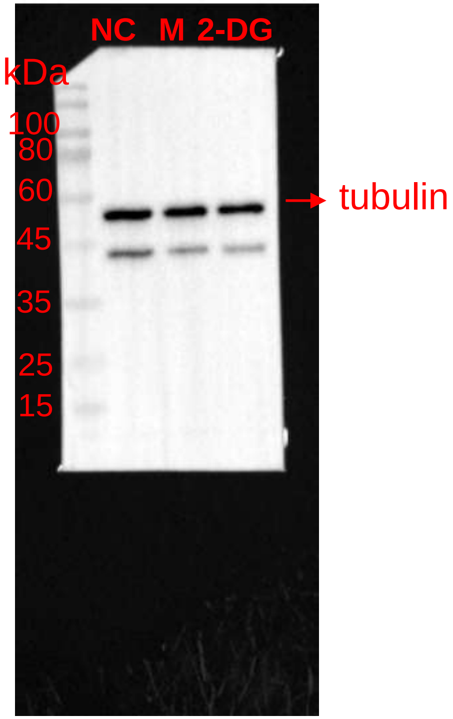

Figure 3i Western blot

β-actin (43kDa)

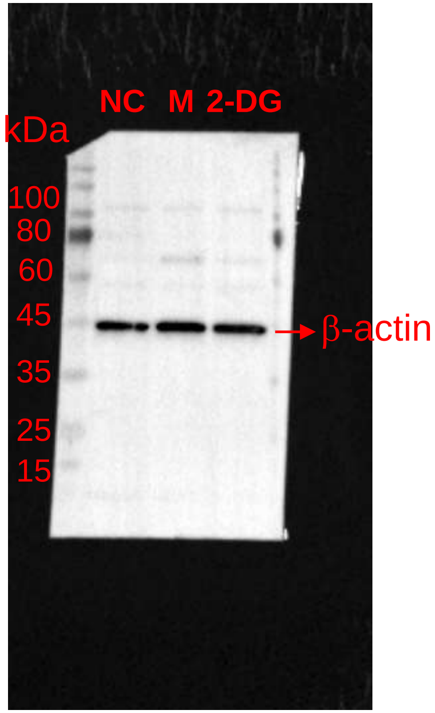

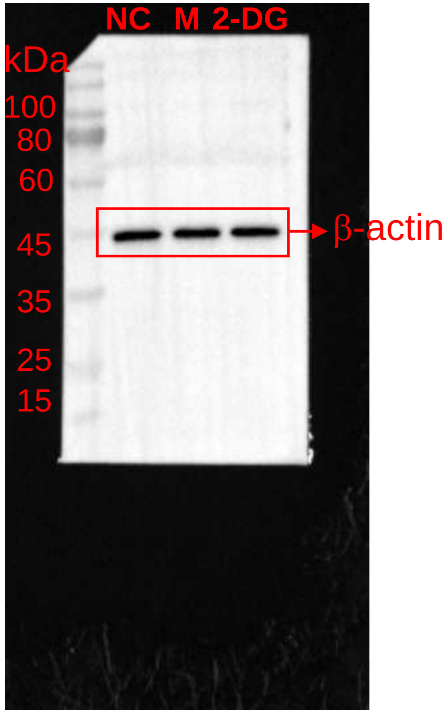

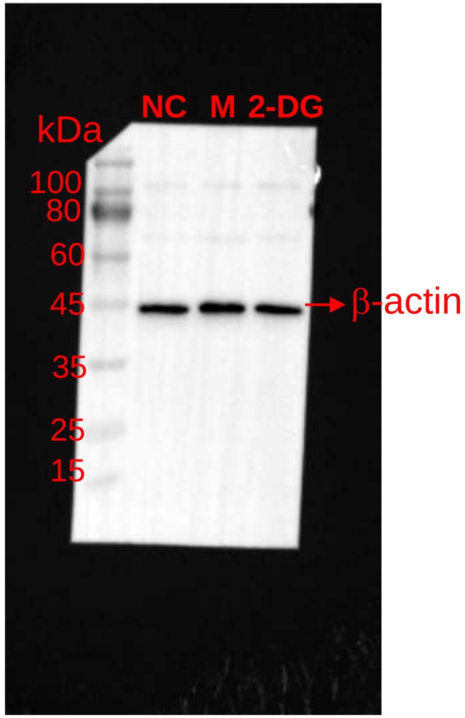

Figure 4e Western blot

HK2(102kDa)

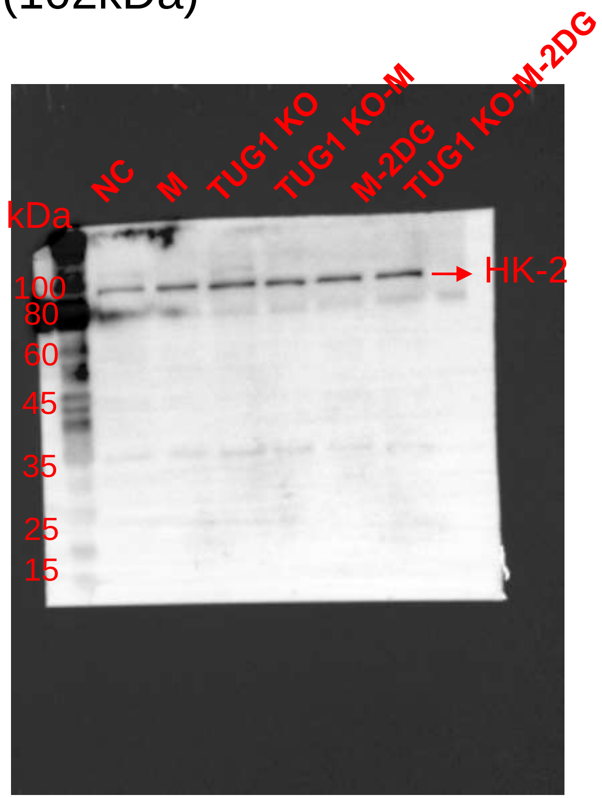

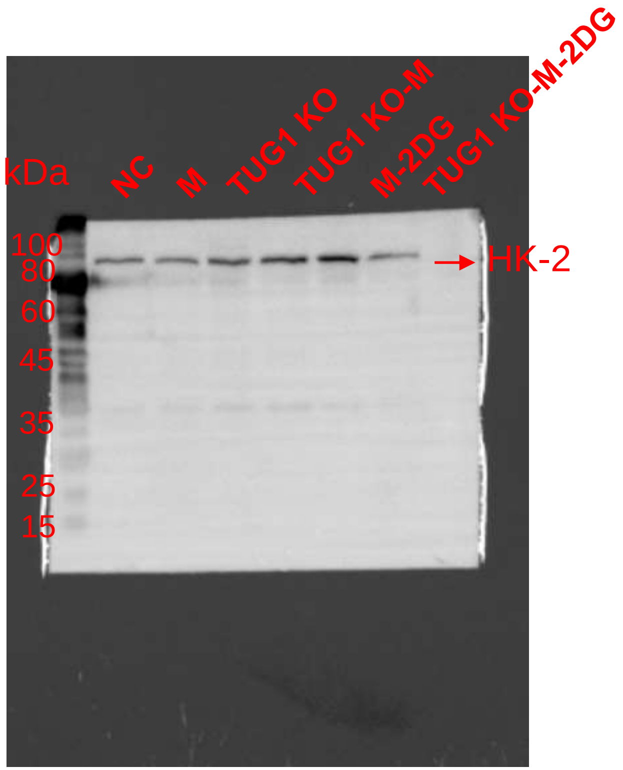

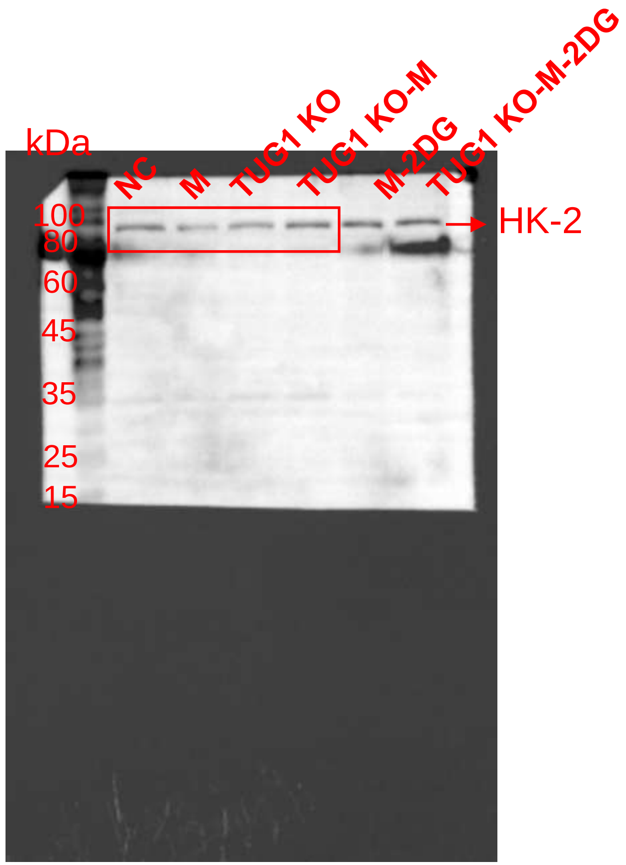

Figure 4e Western blot

G6PD(59kDa)

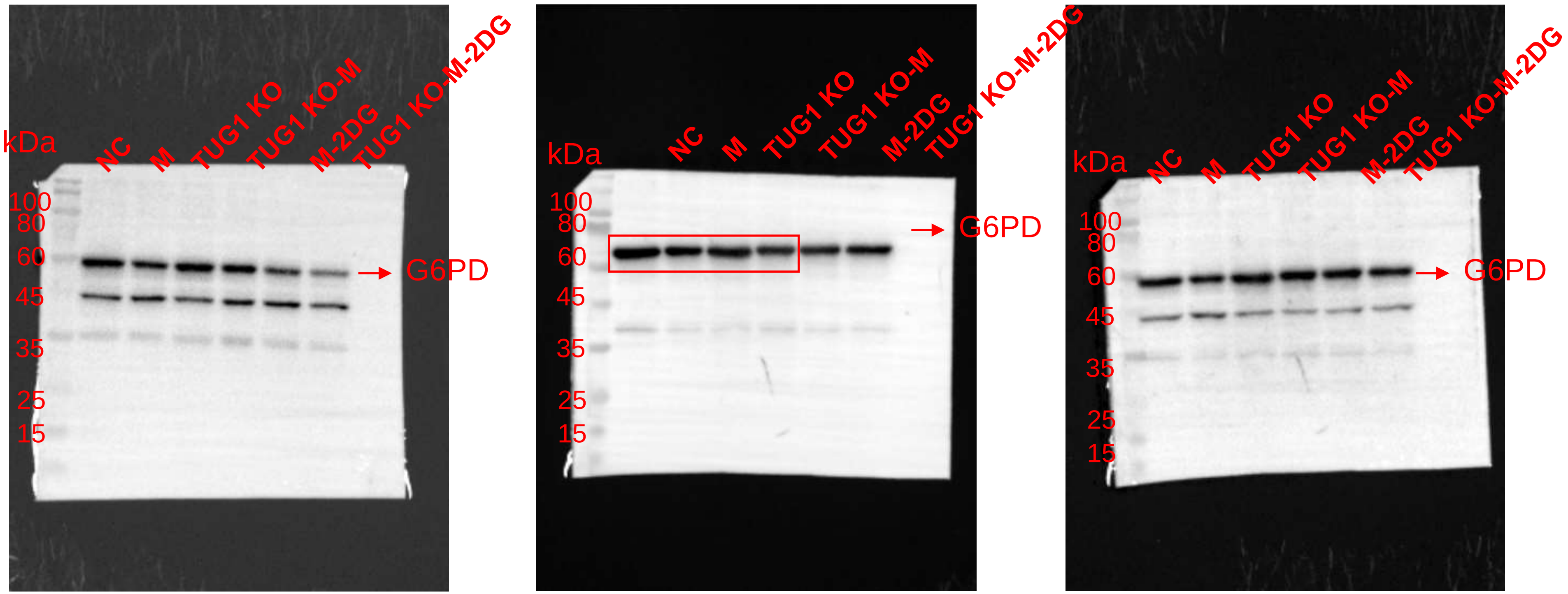

Figure 4e Western blot

PDH(43kDa)

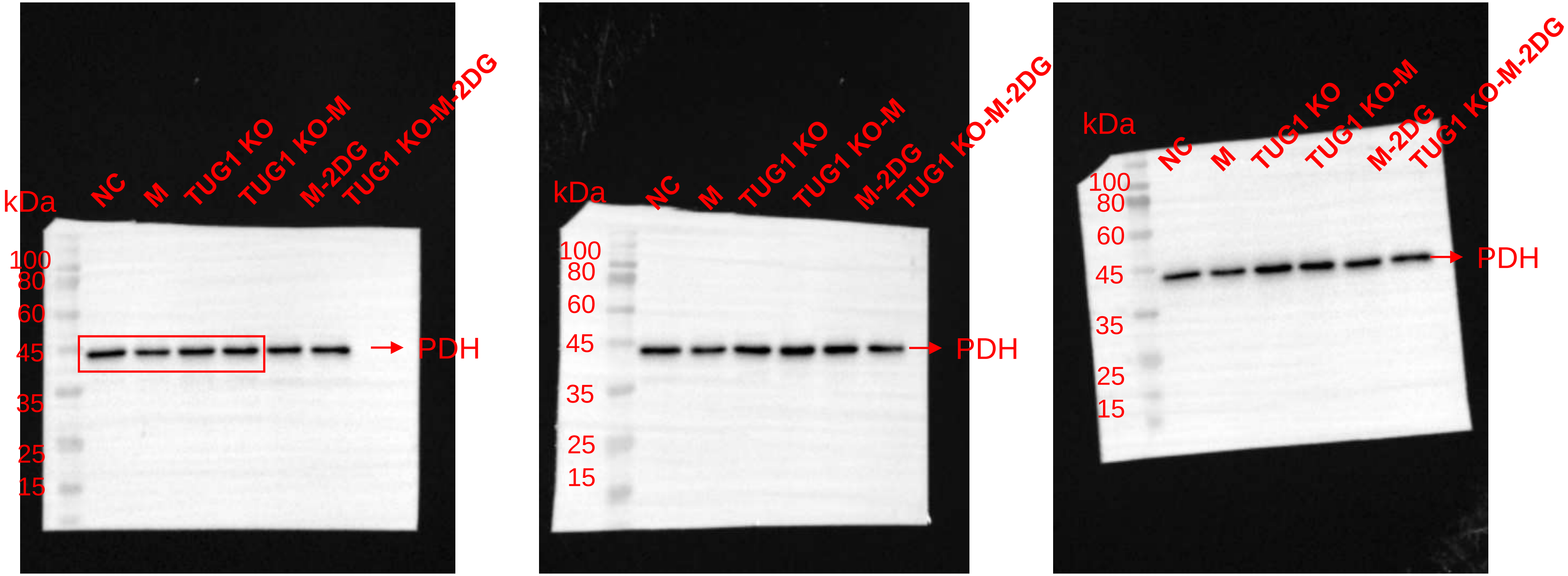

Figure 4e Western blot

Tubulin(55kDa)

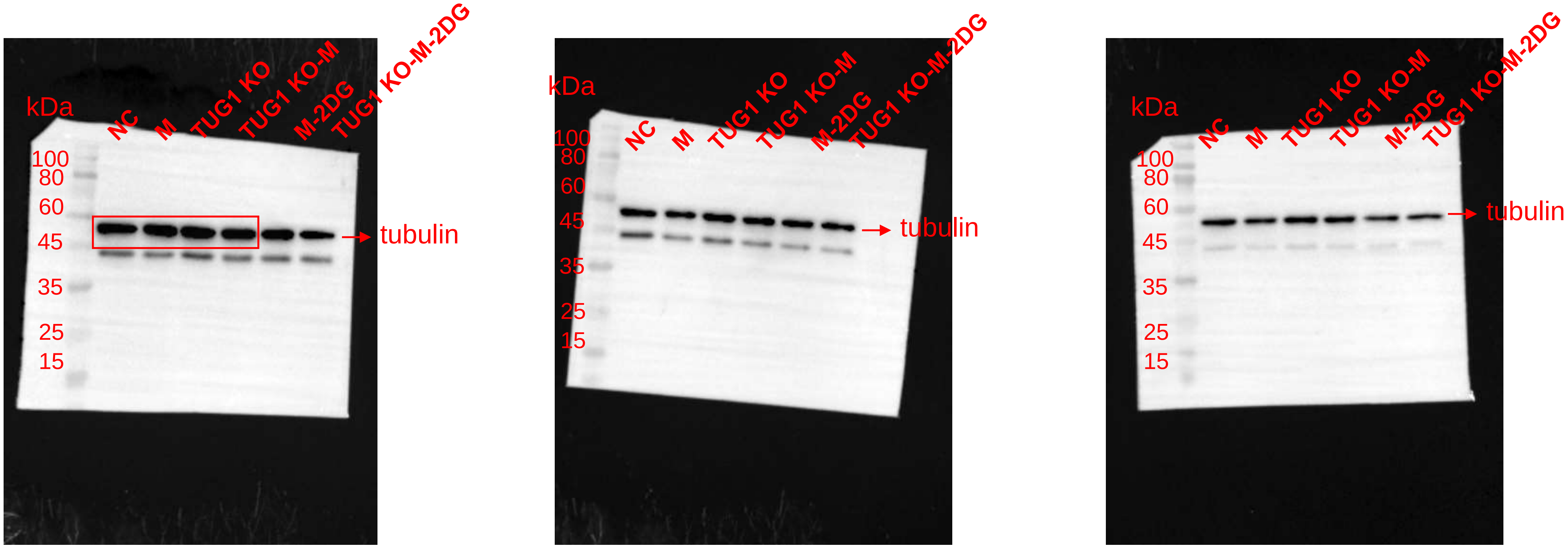

Figure 4e Western blot

β-actin (43kDa)

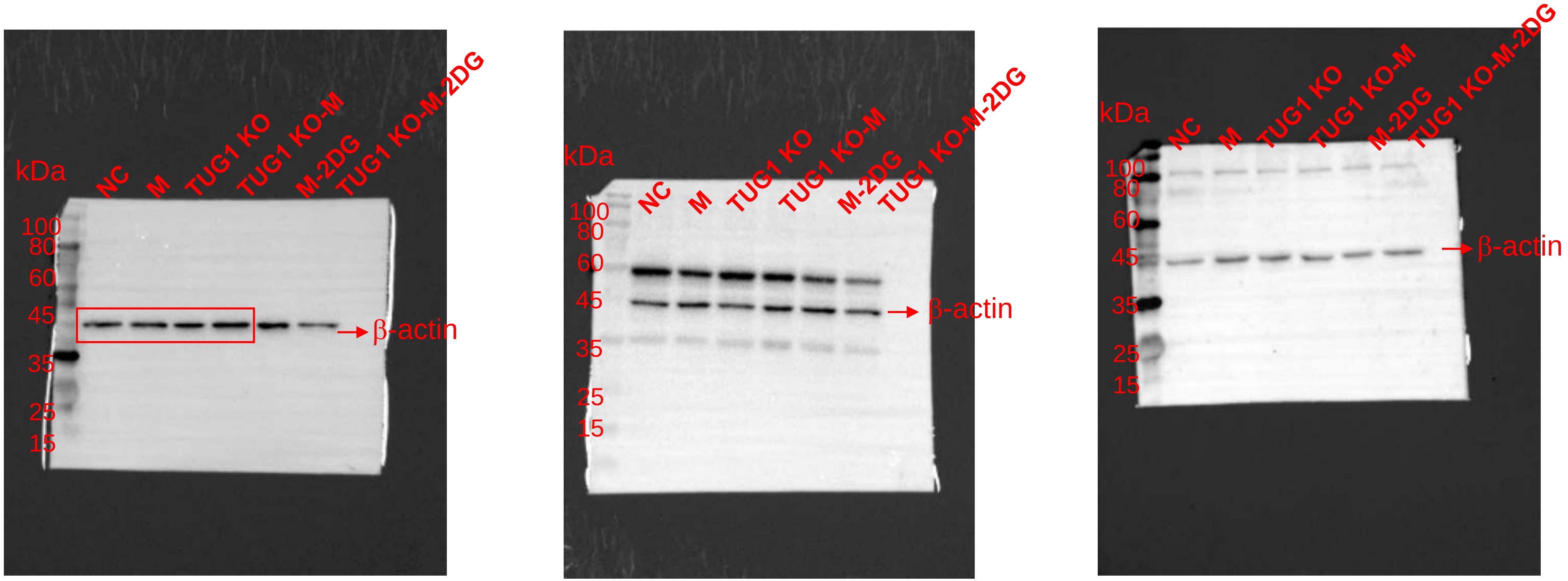

Figure S1i Western blot

HK2(102kDa)

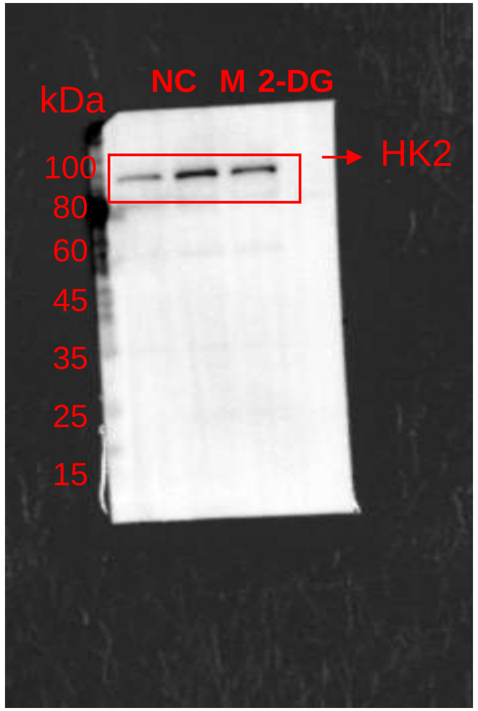

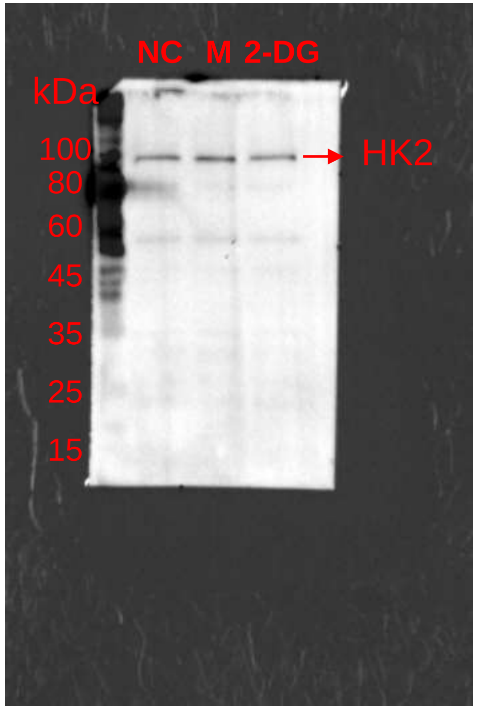

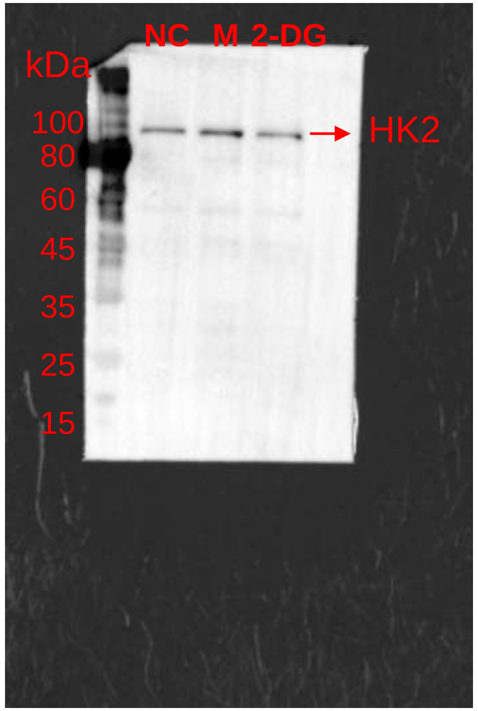

Figure S1i Western blot

G6PD(59kDa)

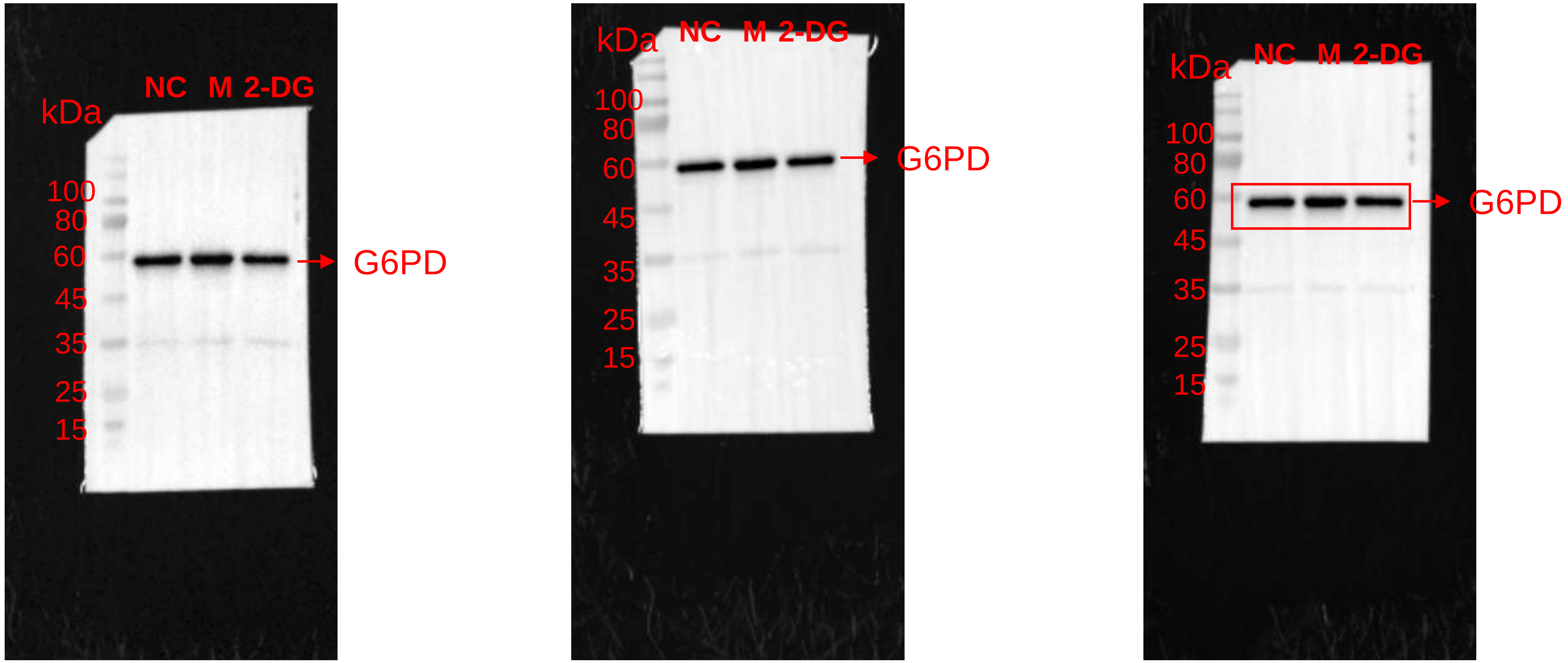

Figure S1i Western blot

PDH(43kDa)

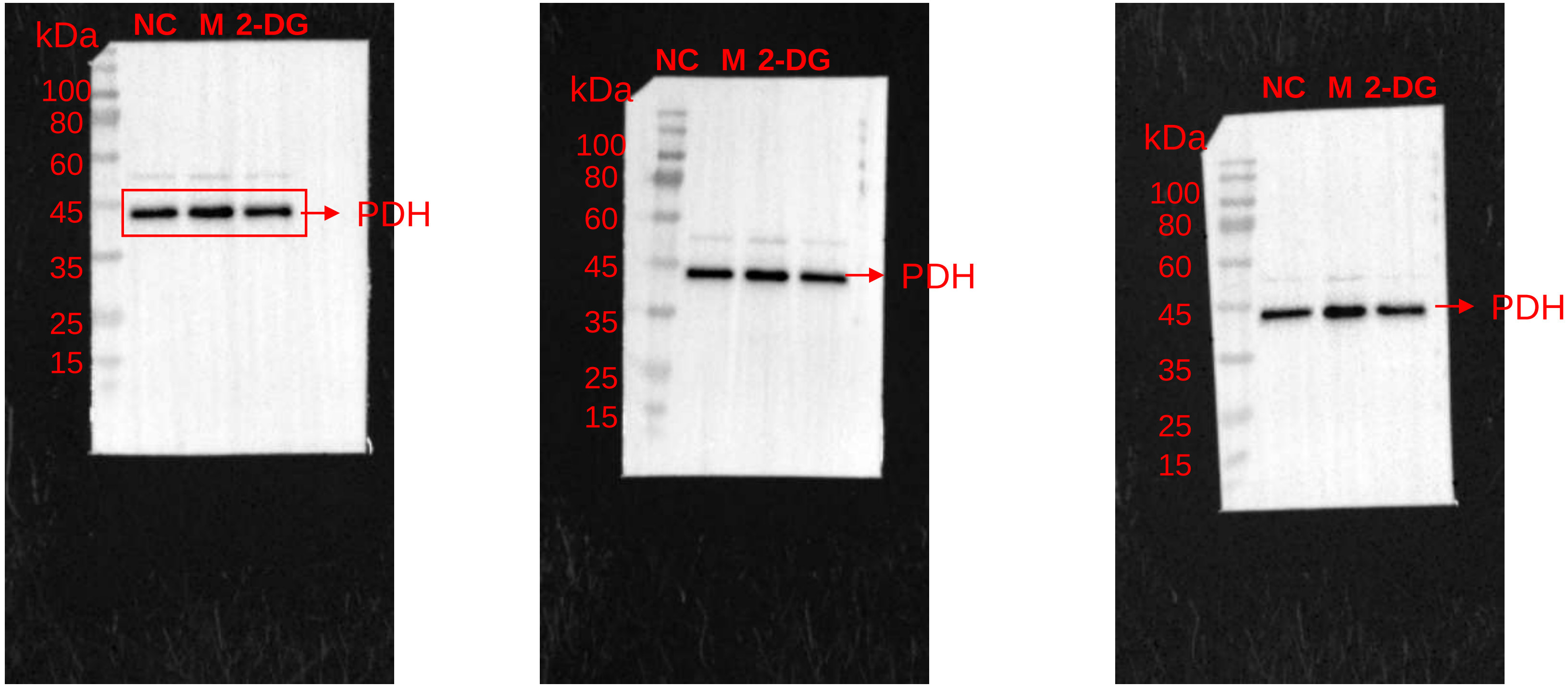

Figure S1i Western blot

Tubulin(55kDa)

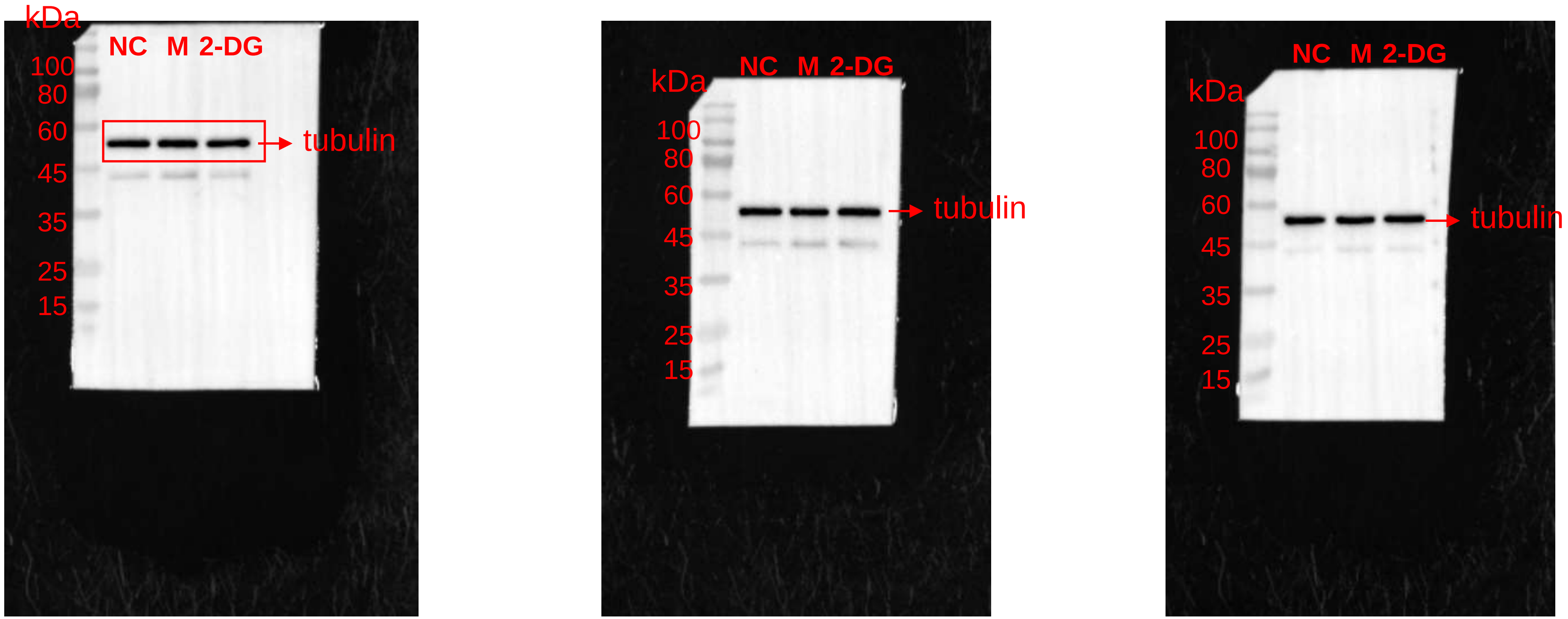

Figure S1i Western blot

β-actin (43kDa)

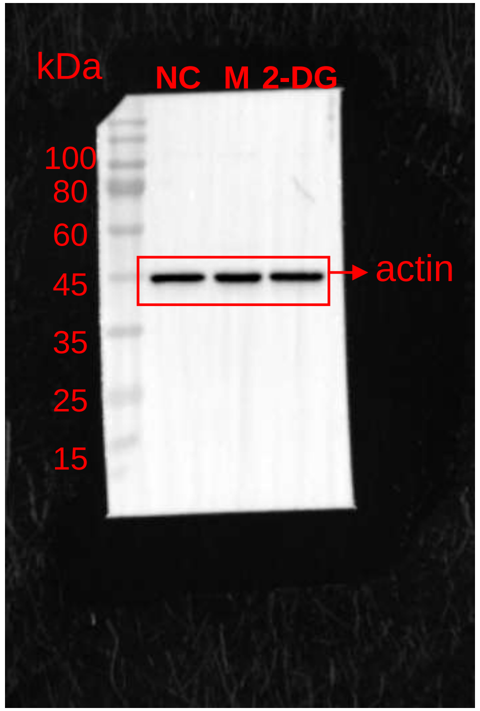

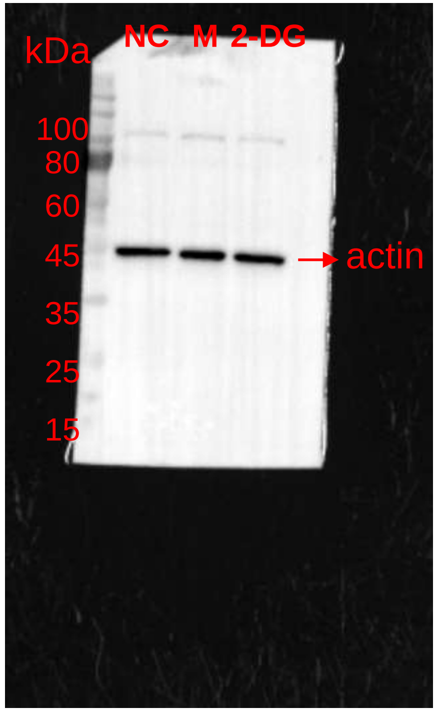

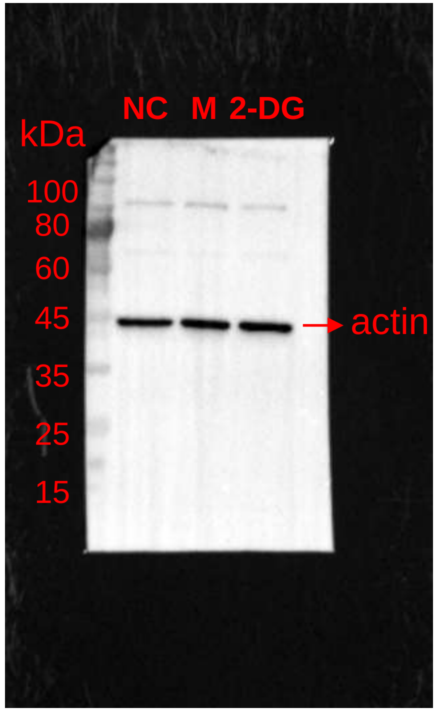

Supplement: Supplementary file 5 — Supplementary Information 5. [file 41598_2024_62966_MOESM5_ESM.pdf]
